# Pharmacokinetic and molecular docking studies to pyrimidine drug using Mn_3_O_4_ nanoparticles to explore potential anti-Alzheimer activity

**DOI:** 10.1038/s41598-024-65166-2

**Published:** 2024-07-04

**Authors:** Wesam S. Shehab, Hend A. Haikal, Doaa A. Elsayed, Ahmed F. EL-Farargy, Abdel-Rahman B. A. El-Gazzar, Gehan T. El-Bassyouni, Sahar M. Mousa

**Affiliations:** 1 Department of Chemistry, Faculty of Science, Zagazig University, Zagazig, 44519 Egypt; 2 Organic Chemistry Department, National Research Centre, 33 El-Buhouth St., Dokki, Cairo, 12622 Egypt; 3 Refractories, Ceramics and Building Materials Department, National Research Centre, 33 El-Buhouth St., Dokki, Cairo, 12622 Egypt; 4 Inorganic Chemistry Department, National Research Centre, 33 El-Buhouth St., Dokki, Cairo, 12622 Egypt

**Keywords:** Nanoparticles, Anti-alzheimer activity, Mn_3_O_4_-NPs, Pharmacokinetic, Molecular docking studies, Computational biology and bioinformatics, Drug discovery, Chemistry

## Abstract

Alzheimer disease (AD) is the cause of dementia and accounts for 60–80% cases. Tumor Necrosis Factor-alpha (TNF-α) is a multifunctional cytokine that provides resistance to infections, inflammation, and cancer. It developed as a prospective therapeutic target against multiple autoimmune and inflammatory disorders. Cholinergic insufficiency is linked to Alzheimer's disease, and several cholinesterase inhibitors have been created to treat it, including naturally produced inhibitors, synthetic analogs, and hybrids. In the current study, we tried to prepared compounds may also support the discovery and development of novel therapeutic and preventative drugs for Alzheimer's using manganese tetroxide nanoparticles (Mn_3_O_4_-NPs) as a catalyst to generate compounds with excellent reaction conditions. The Biginelli synthesis yields 4-(4-cyanophenyl)-6-oxo-2-thioxohexahydropyrimidine-5-carbonitrile when the 4-cyanobenzaldehyde, ethyl cyanoacetate, and thiourea were coupled with Mn_3_O_4_-NPs to produce compound 1. This multi-component method is non-toxic, safe, and environmentally friendly. The new approach reduced the amount of chemicals used and preserved time. Compound 1 underwent reactions with methyl iodide, acrylonitrile, chloroacetone, ethyl chloroacetate, and chloroacetic acid/benzaldehyde, each of the synthetized compounds was docked with TNF-α converting enzyme. These compounds may also support the discovery and development of novel therapeutic and preventative drugs for Alzheimer's disease. The majority of the produced compounds demonstrated pharmacokinetic features, making them potentially attractive therapeutic candidates for Alzheimer's disease treatment.

## Introduction

Alzheimer disease (AD) causes sixty to eighty percent of dementia cases, it affects millions of individuals and is a neurodegenerative brain illness that often causes memory loss and cognitive impairment^1^. Alzheimer's symptoms include neurodegeneration, decreased brain function, apathy, anxiety, delusions, and depression^2–4^. The scarcity of particular treatments available for Alzheimer's disease emphasizes the need of an accurate diagnosis. Positron Emission Tomography (PET) and Magnetic Resonance Imaging (MRI) are commonly used to diagnose AD. PET employs radiotracers to observe, quantify, and document physiological changes in metabolism, neurotransmitters, blood flow, and other factors^5^. MRI uses high-quality contrast of soft tissues and increased spatial analysis with no harm to the patient^2^. As of now, there is no known effective treatment for Alzheimer's disease, which could be related to the lack of a clearly recognized underlying mechanism. AD is characterized by the formation of extracellular amyloid-beta (Aplaques and neurofibrillary tangles in the intracellular environment), gliosis, synaptic loss, and β inflammation, leading to several hypothesized explanations^6,7^.

Alzheimer's Disease (AD), which affects a vast population globally, is defined by the elderly's loss of memory and learning capacity. Cholinergic insufficiency is linked to Alzheimer's disease, and several cholinesterase inhibitors have been created to treat it, including naturally produced inhibitors, synthetic analogs, and hybrids such as Acetylcholinesterase (AChE) and Beta site amyloid precursor protein cleaving enzyme 1 (BACE1).

Acetylcholinesterase (AChE) has rekindled attention as a therapeutic target in Alzheimer's disease (AD) because it improves brain cell function by boosting acetylcholine concentrations^8^. BACE1, an enzyme that cleaves Amyloid Precursor Protein (APP) and produces Amyloid β (Aβ), is a promising target for Alzheimer's Disease (AD) treatment. This β-secretase cleaves not only Amyloid Precursor Protein (APP) and its homologues, but also a small number of substrates such as neuregulin and β subunit of voltage-gated sodium channel, which play a very significant role in the development and proper operation of the brain. Furthermore, various variables associated with both physiological and pathological activities alter BACE1 post-translationally^9^.

Manganese oxides have considerable scientific interest because of their economical and sustainable nature, as well as their important uses in molecular adsorption, ion exchange, catalysis, and water treatment^10^. Manganese oxides have different structures with different oxidation states (2^+^, 3^+^ and 4^+^) that are the most dominant in nature^11^. Hausmannite has a normal spinel structure supplied by the formula, Mn^2+^(Mn^3+^)^2^O_4_ in which both the Mn^2+^ and Mn^3+^ ions conquer the tetrahedral and octahedral sites, respectively, with tetragonal distortion of the Mn^3+^ ions due to Jahn–Teller phenomenon^12,13^. Trimangnese tetraoxide nanoparticles (Mn_3_O_4_) are one of the significant nanoparticles that contain numerous crystal structures formed up of octahedral MnO_6_ were manganese dioxide (MnO_2_), dimanganese trioxide (Mn_2_O_3_), and trimangnese tetraoxide nanoparticles (Mn_3_O_4_)^14^. Mn_3_O_4_ has potential for selective oxidation catalysis^15^. In this study, we outline the preparation of Mn_3_O_4_ nanoparticles using a precursor of manganese nitrate by deployment of a precipitation technique. The processed Mn_3_O_4_ powder nanoparticles were identified by X-ray powder diffraction (XRD), high-resolution transmission electron microscopy (HR-TEM), selected area electron diffraction (SAED) and field emission scanning electron microscopy (FE-SEM) equipped with energy dispersive X-Ray analysis (EDX)^16^. The Biginelli synthesis, which produces pyrimidine compounds, has been extensively studied in recent decades, particularly because of the therapeutic properties of the resulting compounds, which include calcium channel blockers, anticancer, antiviral, antimicrobial, anti-inflammatory, and antioxidant compounds^17,18^.

According to Meng et al., the atomic-level interaction between a ligand/drug and a protein is determined via molecular docking experiments^19^. This information enables us to describe the behavior of our compounds in the binding sites of targets and to provide an explanation of basic biochemical processes. Using Molecular Operating Environment (MOE) program (ver.2022), each of the synthetized compounds was docked with TNF-α converting enzyme^20^. These compounds could also help in the discovery and creation of novel therapeutic and defensive drugs for Alzheimer's disease. Before a chemical can be considered a therapeutic molecule, it must first be validated by a number of parameters, including potential toxicity, absorption, distribution, metabolism, and excretion (ADME) properties, and pharmacokinetic characteristics. We used a number of bioinformatics methods to verify each of our prepared molecules^21^.

## Results and discussion

### Characterization of Mn_3_O_4_ nanoparticles

#### XRD analysis

**Figure 1:**
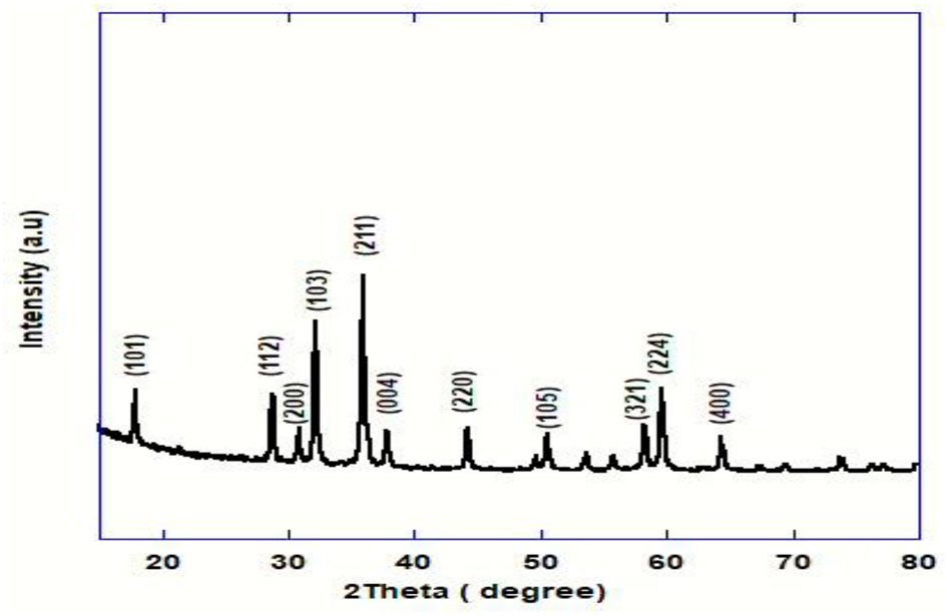
XRD pattern of Mn_3_O_4_-NPs.

The XRD pattern of crystalline sample exhibits diffraction peaks for Hausmanite structure (Mn_3_O_4_) (Fig. 1). The acquired diffraction peak positions are matched with the standard value and are found to be compatible with [(JCPDS 24-0734)] which is related to tetragonal single phase of (Mn_3_O_4_). The lattice parameters of the tetragonal unit cell were found to be (a = b = 5.76 Å and c = 9.46 Å) which is consistent with prior reports^22^. No other impurity peaks were observed in the XRD pattern, indicating that no other manganese oxide formulations were detected.

#### Morphological and elemental analyses

**Figure 2:**
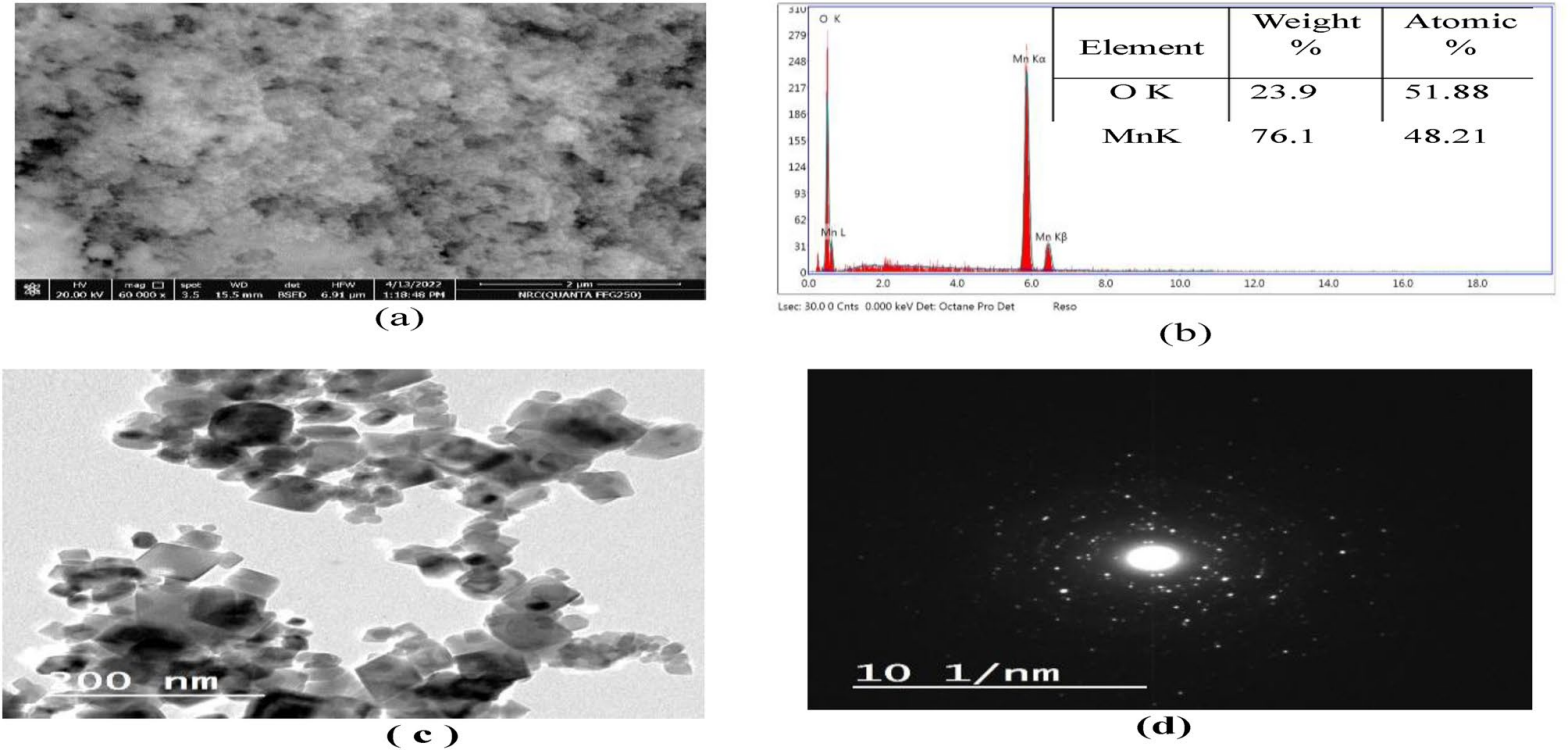
SEM image (**a**) with EDX spectrum (**b**) and TEM image (**c**) with diffraction pattern of Mn_3_O_4_-NPs (**d**).

The morphology, particle size, and crystallinity of the Mn_3_O_4_ nanoparticles have been investigated using FE-SEM and TEM micrographs. Figure 2a depicts FE-SEM image of Mn_3_O_4_-NP. The particles were found to be highly agglomerated but homogeneous in size, and their shape was either spherical or cubic^23^. The EDX results shown in Fig. 2b highlighted the incidence of solely Mn and O elements, indicating the exceptional clarity of the synthesized sample. Figure 2c illustrates the typical TEM image of manganese oxide nanostructure (Mn_3_O_4_)^24^. It shows that the sample consisted mainly of cubic particles and a few spherical structures with particle size found to be in the range 15–70 nm. This observation is consistent with the SEM results^25^. The SAED pattern in Fig. 2d illustrates the polycrystalline nature of the Mn_3_O_4_ nanoparticles^26^.

### Chemistry

**Figure 3:**
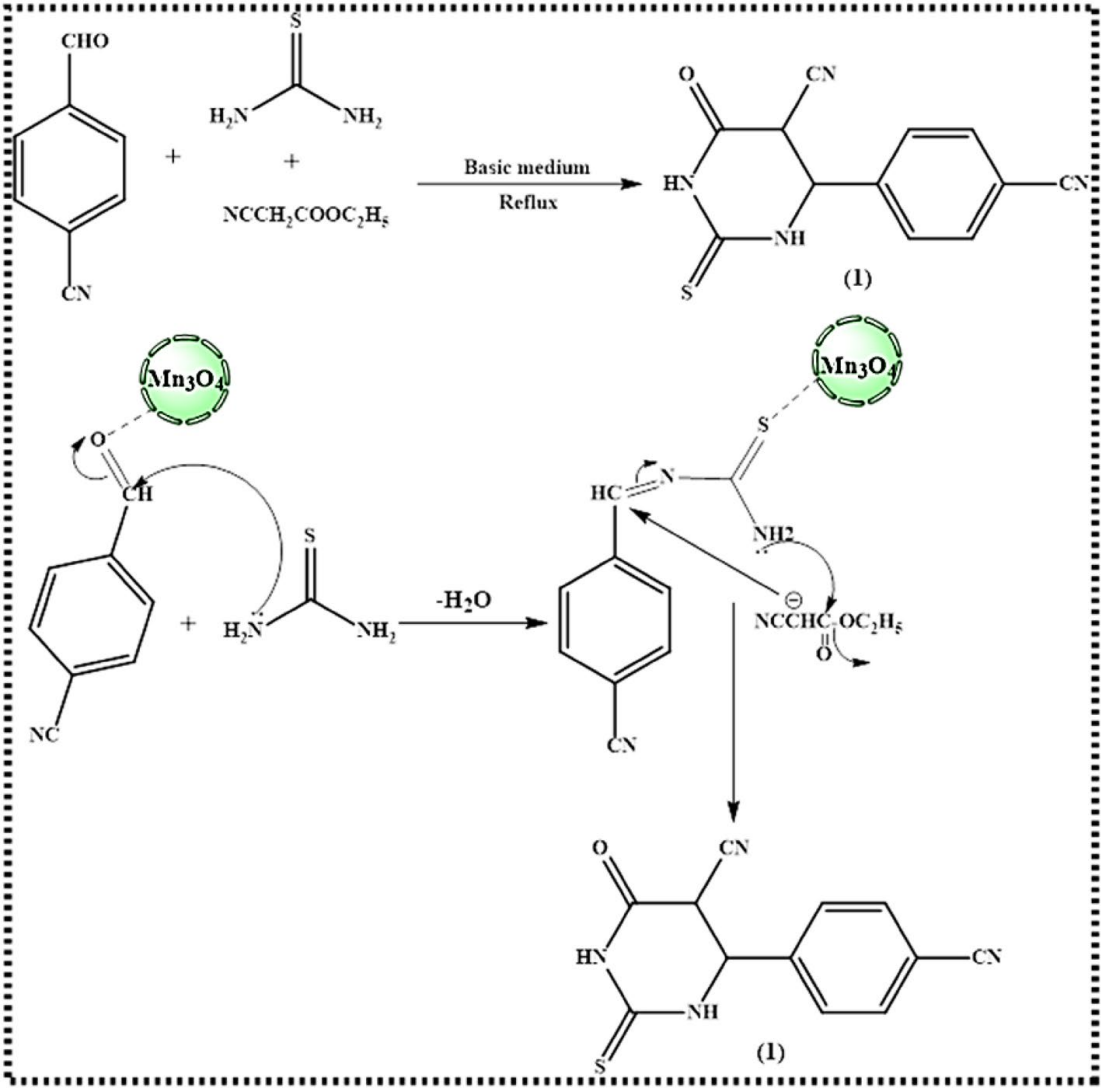
Synthesis of 4-(4-cyanophenyl)-6-oxo-2-thioxohexahydropyrimidine-5-carbonitrile.

Depending on the pyrimidine moiety, novel heterocyclic compounds were synthesized. Two approaches were used to synthesize the starting material 4-(4-cyanophenyl)-6-oxo-2-thioxohexahydropyrimidine-5-carbonitrile (1). The conventional procedure comprises the reaction of 4-cyanobenzaldehyde with thiourea, or ethyl cyanoacetate, in ethanol under reflux conditions along with few drops of triethylamine. In order to obtain compound (1) with optimal reaction conditions, table (1), the three components' reaction in the presence of Mn_3_O_4_ nanoparticles (Mn_3_O_4_-NPs) as a catalytic amount in ethanol under reflux was recorded (Fig. 3). The structure 1 reviewed was clarified with the use of infrared (IR), proton, and carbon-13 nuclear magnetic resonance (^1^H-NMR and ^13^C-NMR). An absorption band at 1721 cm^−1^ ascribed to the C=O group, an absorption band at 2224 cm^−1^ attributable to the CN group, a C=S band at 1270 cm^−1^, and an absorption band across the range 3343–3521 cm^−1^ recognized to the 2NH groups were all seen in the IR spectra. The ^1^H NMR spectra displayed two doublet signals at 8.05 and 8.15 because of the CH aromatic. The ^13^C NMR spectrum revealed two signals at ( in ppm) 115.06, 118.14 owing to 2 CN groups, a signal at 161.22 due to C=O group, and a signal at 183.81 due to C=S group. The spectra also revealed two singlets’ signals at 8.17 and 8.49 due to 2 NH groups.

Due to the low yield of compounds 1, 2 and 5 in traditional method, the reaction was optimized using Mn_3_O_4_-NPs. It became apparent that the finest features were attained when the Mn_3_O_4_-NPs was elaborated in the reaction as shown in Table 1. The tabulated results showed that Mn_3_O_4_-NPs achived best results in time and yield.

**Table 1:** Optimization of the Reaction Conditions.

Compound	Solvent	Catalyst (base)	Temperature (°C)	Time (h)	Yield (%)
**1**	Ethanol	TEA	Reflux	3	35
**1**	Ethanol	Mn_3_O_4_	Reflux	1	65
**2**	Ethanol	NaOEt	Reflux	3	60
**2**	Ethanol	Mn_3_O_4_	Reflux	1.30	80
**5**	Ethanol	Pyridine	Reflux	5	45
**5**	Ethanol	Mn_3_O_4_	Reflux	2	70

**Figure 4:**
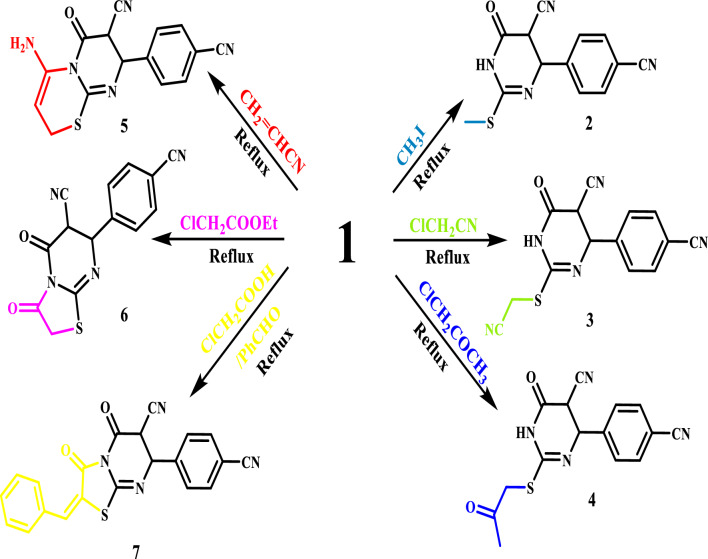
Synthesis of pyrimidine derivatives.

Treating (1) with methyl iodide in ethanolic sodium ethoxide resulted in the production of the corresponding methyl-thio derivative 2, which was used to introduce a suitable leaving group. Moreover, Mn_3_O_4_-NPs are used as a basic environment for the alkylation reaction. Cyanomethylation with chloroacetonitrile in ethanolic sodium ethoxide produced 2-((cyanomethyl)thio)-4-(4-cyanophenyl)-6-oxo-1,4,5,6-tetrahydropyrimidine-5-carbonitrile (3). 4-(4-cyanophenyl)-6-oxo-2-((2-oxopropyl)thio)-1,4,5,6-tetrahydropyrimidine-5-carbonitrile (4) was produced by basic alkylation of compound (1) with chloroacetone (Fig. 2). 4-amino-8-(4-cyanophenyl)-6-oxo-7,8-dihydro-2H,6H-pyrimido[2,1-b][1,3]thiazine-7-carbonitrile was obtained by treating (1) with acrylonitrile in pyridine (5). Compound (5) is also generated when acrylonitrile and thiol compound (1) react in the presence of Mn_3_O_4_-NPs in ethanol as a catalytic quantity. Under standard circumstances, compound (1) reacted with ethyl chloroacetate to produce the thiazolo[3,2-a]pyrimidine-6-carbonitrile derivative (6). On the other hand, the benzylidene derivative of thiazolo[3,2-a]pyrimidine-6-carbonitrile (7) was produced by compound (1) reacting with chloroacetic acid and benzaldehyde under reflux in a combination of glacial acetic acid and acetic anhydride (Fig. 4). Based on their spectrum data, compounds 2, 3, 4, 5, 6, and 7's structures were verified (experimental portion).

**Figure 5:**
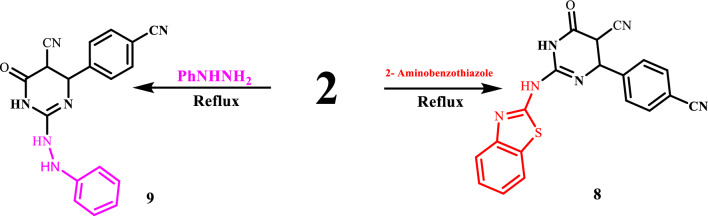
Synthesis of isolated pyrimidine derivatives.

The structure of 2 is supported by the fact that it can be converted into 2-(benzo[d]thiazol-2-ylamino)-4-(4-cyanophenyl)-6-oxo-1,4,5,6-tetrahydropyrimidine-5-carbonitrile (8) by reacting with 2-amino benzothiazole in the presence of a small amount of triethylamine, and it can also be converted into 4-(4-cyanophenyl)-6-oxo-2-(2-phenylhydrazine)-1,4,5,6 tetrahydropyrimidine-5-carbonitrile (9) by reacting with phenylhydrazine under conditions that are similar, Fig. 5. Compound 2's S-CH_3_ methyl group signal vanished, according to ^1^H NMR spectra, around 2.53 ppm. A few drops of triethylamineafforded2-(benzo[d]thiazol-2-ylamino)-4-(4-cyanophenyl)-6-oxo-1,4,5,6-tetrahydropyrimidine-5-carbonitrile are added to compound 2 in reaction with 2-amino benzothiazole (8). Compound (2) interacted with phenylhydrazine under comparable experimental circumstances, producing 4-(4-cyanophenyl)-6-oxo-2-(2-phenylhydrazine)-1,4,5,6-tetrahydropyrimidine-5-carbonitrile (9), as shown in (Fig. 5).

### Insilco-studies

**Figure 6:**
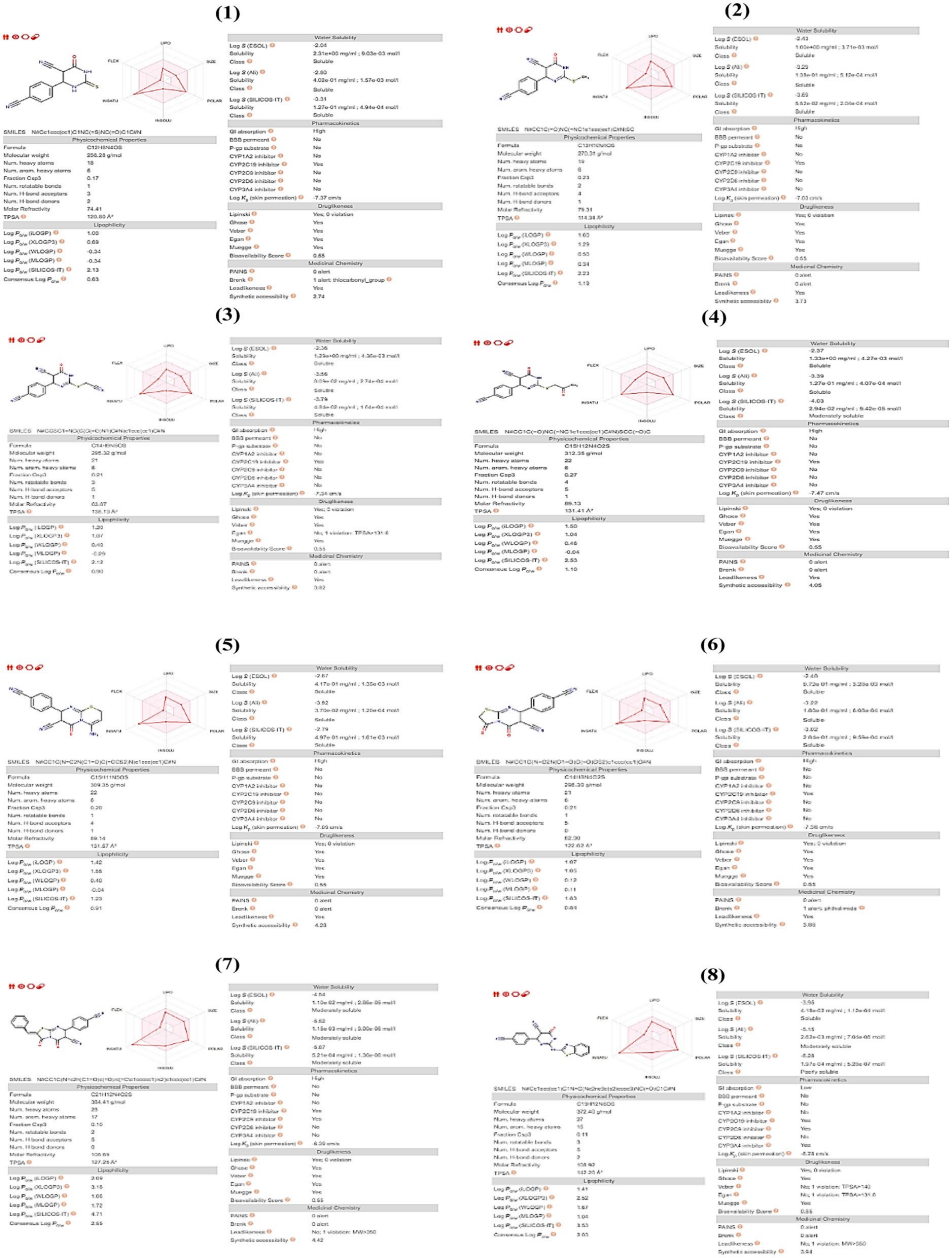
Computed values for prediction parameters of Synthetic compounds.

Analyzing drug-likeness and oral bioavailability of synthetic compounds utilizing Swiss ADME web tools Early in the drug development process, it is critical to assess the pharmacokinetic features of candidate drugs. Drugs must obey the rule of five revolutions (RO5), as revealed by Lipinski in 2004^27^. In Fig. 6, each molecule was tested against anti-Alzheimer to see if it met the RO5 criteria, which included the properties specified in Table 2 and Fig. 6.

**Table 2:** Using Lipinski's rule of five (RO5) of anti-Alzheimer and chosen bioactive substances of synthesized compounds as possible protein inhibitors.

Ligands	Molecular formula	Molecular weight < 500	Log *P* < 5	H-bond donor < 5	H-bond acceptor < 10	Meet RO5
1	C_12_H_8_N_4_OS	258.28	0.63	2	3	0.55
2	C_13_H_10_N_4_OS	270.31	1.19	1	4	0.55
3	C_14_H_9_N_5_OS	395.32	0.90	1	5	0.55
4	C_15_H_12_N_4_O_2_S	312.35	1.10	1	5	0.55
5	C_15_H_11_N_5_OS	309.35	0.91	1	4	0.55
6	C_14_H_8_N_4_O_2_S	296.30	0.84	0	5	0.55
7	C_21_H_12_N_4_O_2_S	384.41	2.55	0	5	0.55
8	C_19_H_12_N_8_OS	372.40	2.03	2	5	0.55
9	C_24_H_22_N_8_O	438.48	2.16	5	5	0.55

### Molecular docking

Pyrimidine derivatives are widely known for being sources of medications for a variety of human problems and have been utilized since ancient times. For our research, we selected 9 synthetic pyrimidine derivatives occurring substances with exceptional antioxidant capabilities that primarily function by scavenging free radical species. Significant information has been acquired recently that points to an increase in oxidative stress in the brain of people with AD. According to Badrul and Ekramu, this could play a part in the pathophysiology of neuron degeneration and death^28^. Therefore, defense and suppression of oxidative stress might be regarded as a crucial factor in the creation of anti-Alzheimer medications.

**Figure 7:**
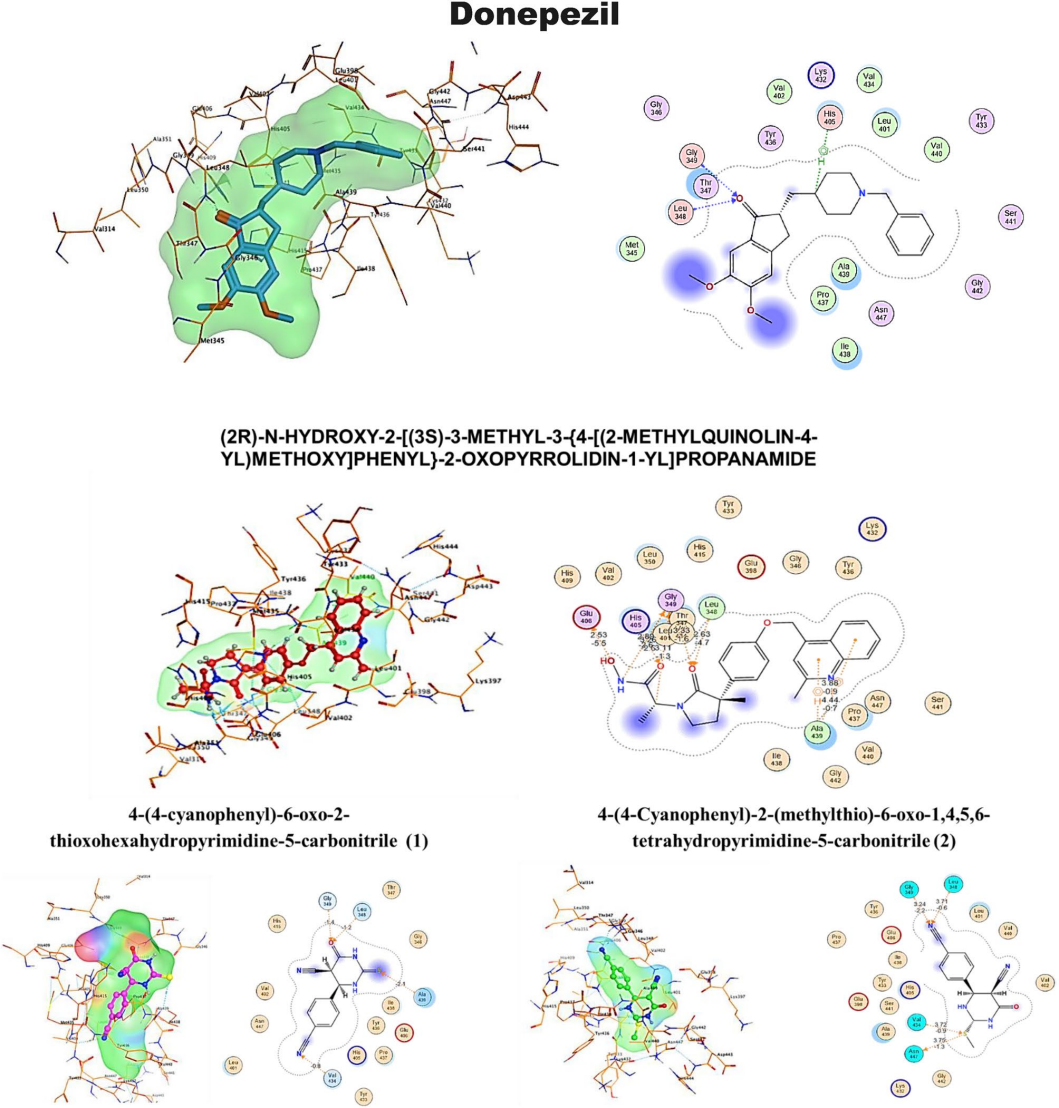

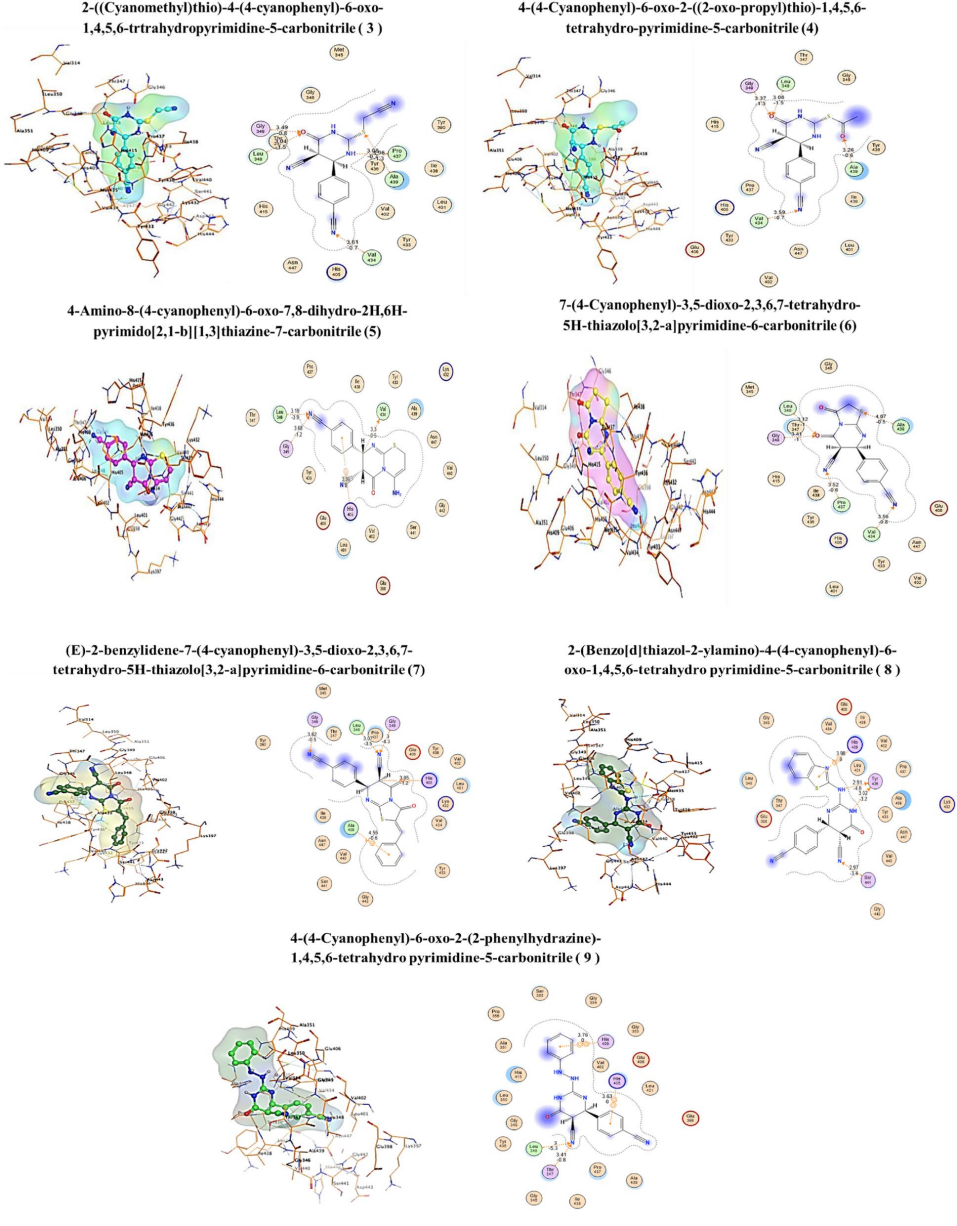
2D, 3D receptor interactions of the promising synthetized compounds against Anti-Alzheimer.

Molecular docking study of the created database was utilized to explore the hypothesized mechanism of action for the newly discovered and synthesized drug candidates as anti-Alzheimer compared to (2R)-n-hydroxy-2-[(3S)-3-methyl-3-{4-[(2-methylquinolin-4-yl)methoxy]phenyl}-2-oxopyrrolidin-1-yl]propenamide as co-crystallized ligand was isolated as a standard reference from TNF-α Converting Enzyme (TACE) in complex with IK682 enzyme (PDB code: 2FV5). This investigation was carried out to get deeper insight into the binding mechanisms of the produced compounds into the protein-binding site of the TACE in association with the enzyme IK682. The co-crystallized ligand was re-docked into the active site consuming the same parameters to confirm the existing docking study at the active site. The best-docked pose's root means square deviation (RMSD) was 2.7122 Å, and the energy score was − 9.6854 kcal/mol, thus validating the docking study with M.O.E. software. In addition, the co-crystallized ligand formed 3 hydrogen bonds with GLY 349 (A), GLY 349 (A), and one with GLU 406 (A), LEU 348 (A) and HIS 405 (A). Although interacted by pi-H with ALA 439 (A) and ALA 439 (A), Fig. 7.

Each of the compounds was docked with Alzheimer disease-associated target individually. These compounds showed very good binding affinity with Alzheimer-associated target. Compounds 3, 5, 6, 7 and 8 showed prefect docking score energy, as shown in Tables 3 and 4.

**Table 3:** The binding scores of the promising compound.

Compound	E-conf	E-place	E-score1	E-refine	E-score2
Ref	69.7962	− 77.8181	− 9.4985	− 61.6467	− 9.6845
**donepezil**	**62**.**0398**	− **120**.**7095**	− **12**.**1517**	− **45**.**5541**	− **8**.**2233**
**1**	− 131.5904	− 58.9069	− 9.6072	− 34.9427	− 6.4697
**2**	− 144.1257	− 71.0433	− 9.753	− 31.8856	− 6.9902
**3**	− 161.7405	− 75.4804	− 10.0410	− 41.8340	− 7.1266
**4**	− 191.3430	− 99.6022	− 9.6838	− 39.2183	− 6.9920
**5**	− 115.9936	− 103.7516	− 11.2020	− 39.0200	− 7.7098
**6**	− 92.8330	− 80.4992	− 10.9414	− 46.7746	− 8.1636
**7**	− 135.4684	− 102.3331	− 12.1820	− 44.6312	− 8.2446
**8**	− 103.9546	− 188.9370	− 10.1013	− 41.3786	− 7.2720
**9**	− 16.1874	− 52.0129	− 9.3119	− 30.8102	− 6.9020

Significant values are in bold.

**Table 4:** The binding scores, RMSD values, ligand, receptor, interactions, distance, interactions with distance and energy for the promising compounds compared to the reference ligand as a reference for Anti-Alzheimer.

Compound	Score (Kcal/mol)	RMSD	Ligand	Receptor			Interactions	Distance (Å)	E (Kcal/mol)
**Ref**	− 9.6854	2.7122	C	O	GLY	349 (A)	H-donor	3.11	− 1.3
			N2	O	GLY	349 (A)	H-donor	2.89	− 2.6
			O	OE1	GLU	406 (A)	H-donor	2.53	− 5.5
			O3	N	LEU	348 (A)	H-acceptor	2.63	− 4.7
			O3	N	GLY	349 (A)	H-acceptor	3.33	− 1.6
			O4	NE2	HIS	405 (A)	H-acceptor	3.26	− 2.6
			6-ring	CA	ALA	439 (A)	pi-H	3.88	− 0.9
			6-ring	CA	ALA	439 (A)	pi-H	4.44	− 0.7
**donepezil**	− **8**.**22334**	**1**.**2173**	**O20**	**N**	**LEU**	**348 (A)**	**H-acceptor**	**3**.**02**	− **1**.**2**
			**O20**	**N**	**GLY**	**349 (A)**	**H-acceptor**	**3**.**47**	− **1**.**1**
			**C24**	**5-ring**	**HIS**	**405 (A)**	**H-pi**	**3**.**62**	− **0**.**7**
**1**	− 6.4697	1.4346	S	N	ALA	439 (A)	H-acceptor	4.17	− 2.1
			N	CA	VAL	434 (A)	H-acceptor	3.55	− 0.8
			O	N	LEU	348 (A)	H-acceptor	3.15	− 1.2
			O	N	GLY	349 (A)	H-acceptor	3.25	− 1.4
**2**	− 6.9902	1.8001	S	O	ASN	447 (A)	H-donor	3.75	− 1.3
			S	CA	VAL	434 (A)	H-acceptor	3.72	− 0.9
			N	N	LEU	348 (A)	H-acceptor	3.71	− 0.6
			N	N	GLY	349 (A)	H-acceptor	3.24	− 2.2
**3**	− 7.1266	1.3345	N	O	PRO	437 (A)	H-donor	3.08	− 0.7
			O	N	LEU	348 (A)	H-acceptor	3.04	− 1.5
			O	N	GLY	349 (A)	H-acceptor	3.49	− 0.8
			S	N	ALA	439 (A)	H-acceptor	3.96	− 1.3
			N	CA	VAL	434 (A)	H-acceptor	3.61	− 0.7
**4**	− 6.9920	0.8138	O	N	LEU	348 (A)	H-acceptor	3.08	− 1.5
			O	N	GLY	349 (A)	H-acceptor	3.37	− 1.3
			O	N	ALA	439 (A)	H-acceptor	3.26	− 0.6
			N	CA	VAL	434 (A)	H-acceptor	3.59	− 0.7
**5**	− 7.7098	0.8417	C	O	VAL	434 (A)	H-donor	3.30	− 0.5
			N	N	LEU	348 (A)	H-acceptor	3.18	− 3.9
			N	N	GLY	349 (A)	H-acceptor	3.68	− 1.2
			6-ring	5-ring	HIS	405 (A)	pi–pi	3.96	− 0.0
**6**	− 8.1636	1.3054	O	N	LEU	348 (A)	H-acceptor	3.12	− 1.0
			O	N	GLY	349 (A)	H-acceptor	3.41	− 1.0
			S	N	ALA	439 (A)	H-acceptor	4.07	− 0.5
			N	CA	PRO	437 (A)	H-acceptor	3.52	− 0.6
			N	CA	VAL	434 (A)	H-acceptor	3.56	− 0.8
**7**	− 8.2446	1.4969	N	N	LEU	348 (A)	H-acceptor	3.07	− 3.5
			N	N	GLY	349 (A)	H-acceptor	3.00	− 4.3
			N	CA	GLY	346 (A)	H-acceptor	3.62	− 0.5
			C	5-ring	HIS	405 (A)	H-pi	3.95	− 1.2
			6-ring	CA	ALA	439 (A)	pi-H	4.55	− 0.6
**8**	− 7.2720	0.9042	N	O	TYR	436 (A)	H-donor	3.02	− 3.2
			N	O	TYR	436 (A)	H-donor	2.91	− 4.8
			N	N	SER	441 (A)	H-acceptor	2.97	− 3.4
			5-ring	5-ring	HIS	405 (A)	pi–pi	3.98	− 0.0
**9**	− 6.9020	1.7828	N	CA	THR	347 (A)	H-acceptor	3.41	− 0.8
			N	N	LEU	348 (A)	H-acceptor	3.00	− 5.3
			6-ring	5-ring	HIS	409 (A)	pi–pi	3.76	− 0.0
			6-ring	5-ring	HIS	405 (A)	pi–pi	3.63	− 0.0

Significant values are in bold.

Donepezil was re-docked into the active site consuming the same parameters to confirm the existing docking study at the active site. The best-docked pose's root means square deviation (RMSD) was 1.2173 Å, and the energy score was − 8.223 kcal/mol, Compounds 5, 6, 7 and 8 showed prefect docking score energy compared with commercial drug Donepezil.

## Experimental section

### Materials and methods

High-grade materials were used to synthesize the target compounds. Chemicals were obtained from Sigma-Aldrich (Taufkirchen, Germany). Solvents were provided by Sigma-Aldrich Company. The manufactured Mn_3_O_4_ nanoparticles were provided by the National Research Centre (NRC) by means of precipitation technique. The nanoparticle nature and crystallinity were evaluated using a high-resolution transmission electron microscope (HR-TEM) [JEM-2100, Tokyo, Japan]. The liquefied solution of the particles was drop-casted onto a carbon-coated copper grid and air-dried at ambient temperature in advance of being examined under a microscope. X-ray diffraction (XRD) was used to identify the phase of prepared dried powder using Bruker Axs diffractometer model (D8 Advance, Germany) in the range of 2θ = 15°–80°. Melting points were measured without correction using a digital Electrothermal IA 9100 Series instrument from Cole-Parmer (Beacon Road, Stone, Staffordshire, ST15 OSA, UK). A PerkinElmer CHN 2400 was exploited for C, H, and N analyses. IR spectra were recorded from 4000 to 400 cm^−1^ using FT-IR 460 PLUS utilizing KBr wafers. The ^1^H and ^13^C-NMR spectra were verified by means of a Bruker 800 MHz, NMR Spectrometer, using DMSO-d_6_ as a solvent, and chemical shifts were stated in δ (ppm) at King Abdullah University of Science and Technology (KAUST). The scores obtained after the molecular docking technique were calculated and reported using the Molecular Operating Environment (MOE) program (2022).

### Preparation of Mn_3_O_4_-NPs

To prepare a (0.4 M) solution, 10 g of manganese nitrate (Mn(NO_3_)_2_·4H_2_O—BHD Laborator, Suppliers, 99%) was dissolved in 500 mL of distilled water. A (2 M) solution of NaOH was dripped into the manganese nitrate solution with stirring at room temperature until the pH of the solution reached 10. At this pH value, the solid precipitation appears white and then turns brown after a few seconds. The chemical reaction continued for two hours; the precipitate was kept overnight to simmer down. Brownish precipitate was then washed several times using distilled water to discard the excess of the NaOH followed by filtration and drying at 100 °C for 4 h. The dried clean brown precipitate was kept in a desiccator up to its use.

### Characterization

The processed Mn_3_O_4_-NPs prepared by the precipitation technique are designated by a number of approaches. XRD is accomplished by [Bruker D_8_ advance diffractometer, Germany] with Cu Kα radiation (λ = 1.5406 Å) to study the crystallinity of the material. Microscopic examination such as field emission scanning electron microscope (FE-SEM, model FEJ Quanta 250 Fei, Netherlands) supplemented with Energy Dispersive X-Ray Analysis (EDX) was used to study the size and surface structure of the as-prepared Mn_3_O_4_-NPs. The samples’ surface was coated with a thin layer of gold through a [S150A sputter coater, Edwards, England] under 0.1 Torr, vacuum 1.2 kV voltage and 50 mA current to develop the scanning of samples. In addition, the nanoparticle nature and crystallinity were experienced by high resolution transmission electron microscope (HR-TEM, Joel model JEM-2100, operating voltage 200 kV, Japan). Aqueous dispersion of the particles was drop-casted onto a copper grid coated with carbon before being air dried at room temperature to be microscopically examined.

#### Chemistry

**4-(4-cyanophenyl)-6-oxo-2-thioxohexahydropyrimidine-5-carbonitrile (1)**

A solution containing 1.13 g of ethyl cyanoacetate, 0.76 g of thiourea, and 1.31 g of 4-cyanobenzaldehyde, together with 10 ml of ethanol and a base consisting of a few drops of triethylamine or nano Mn_3_O_4_ (0.079 g, 1 mmol), was heated under reflux. Following cooling, the solid product was filtered out, cleaned with ethanol, and then recrystallized from ethanol to produce yellow crystals. (yield: 35–65%) m.p. 167–170 °C. IR (KBr, ν, cm^−1^):3343–3521 cm^−1^ (2NH str), 3153–2993 cm^−1^ (CH for aromatic and aliphatic), 2224 cm^−1^ (CN), 1721 cm^−1^(C=O), 1270 cm^−1^(C=S). ^1^H-NMR (DMSO-d_6_, 500 MHz): *δ* = 3.34 (*s*, 1*H*, methane–CH–CN), 4.34 (d,1H, CH(CN)–CH–Ar), 8.05,8.15 (*d*, d, 4*H*, CH Aromatic), and 8.49 ppm (s, 2*H*, 2NH)*. *^13^*C-NMR* (DMSO-d_6_, 500 MHz): *δ* = 39.5, 62.7, 106.09, 114.5, 115.06, 118.14, 131.001 133.01, 135.5, 153.1, 161.2, 183.8 ppm. *MS:mle M*^+^*256.8, M*^+*1*^*257.1, M-S atom 224.8. Anal. Calcd. for C*_*12*_*H*_*8*_*N*_*4*_*OS (256.28):C, 56.24; H, 3.15; N, 21.86; S, 12.51.*

**4-(4-Cyanophenyl)-2-(methylthio)-6-oxo-1,4,5,6-tetrahydropyrimidine-5-carbonitrile (2)**

The reaction mixture was refluxed, allowed to cool, and then poured into cold water. The compound 1 (2.56 g, 10 mmol) and methyl iodide (1.4 g, 10 mmol) were combined in a basic medium [ethanolic sodium ethoxide (0.23 g, 10 mmol / 20 ml ethanol) or nano Mn_3_O_4_ (0.079 g, 1 mmol). Utilizing sodium ethoxide After acidification with hydrochloric acid, the solid result (6 ml, 30% soln.) was filtered off, cleaned with water, dried, and then recrystallized from ethanol to produce a yellow powder (yield: 60–80%) m.p. 297–300 °C. IR (KBr, ν, cm^−1^): 3490 (NH, str), 3083–2858 (CH for aromatic and aliphatic), 2231 (CN), 1663 (C=O). ^1^H-NMR (DMSO-d_6_, 500 MHz): *δ* = 2.53(s, 3H, CH_3_), 3.49 (*s*, 1*H,* methine–CH–CN), 2.69 (*d*, 1*H,* CH(CN)–CH–Ar), 8.05,8.09 (d, 4*H*, CH Aromatic), and 13.943 ppm (s, 1*H*, NH)*.* ^13^*C-NMR* (DMSO-d_6_, 500 MHz): *δ* = 13.39, 39.5, 40.41, 94.19, 113.88, 115.51, 118.25 (2CN), 129.53, 132.59, 139.61, 160.69, 165.72, 167.39 (C = O) ppm*. Anal. Calcd. fo*r *C*_*13*_*H*_*10*_* N*_*4*_*OS (270.06): C, 57.76; H, 3.73; N, 20.73; S, 11.86.*

**2-((Cyanomethyl)thio)-4-(4-cyanophenyl)-6-oxo-1,4,5,6-trtrahydropyrimidine-5 carbonitrile (3)**

After stirring the compound 1 solution (0.256 g, 1 mmol) in ethanolic sodium ethoxide (0.0345 g, 1.5 mmol/5 ml ethanol) for an hour at room temperature, the reaction mixture was added to the chloro acetonitrile (0.09 g, 1.2 mmol). It was then gently heated for five hours, allowed to cool, and then poured into ice water. After acidification with hydrochloric acid (6 ml, 30% soln.), the solid result was filtered off, cleaned with water, dried, and then recrystallized from ethanol to produce a dark brown powder. (Yield: 80%) m.p. over 300 °C. IR (KBr, ν, cm^−1^): 3450 (NH, str), 2235, 2204 (2CN), 1642 (C=O). ^1^H-NMR (DMSO-d_6_, 500 MHz): δ = 2.49 (d, 1H, CH(CN)–CH(Ar), 3.61 (s, 1H, (methane–CH–CN), 4.16 (s, 2H, –S–CH_2_–CN), 7.914, 7.931 (d, 4H, CH Aromatic), and 8.06 (s, 1H, NH) ppm. Anal. Calcd. for C_14_H_9_ N_5_ OS (295.32): C, 56.94; H, 3.07; N, 23.71; S, 10.86.

**4-(4-Cyanophenyl)-6-oxo-2-((2-oxo-propyl)thio)-1,4,5,6-tetrahydro-pyrimidine-5-carbonitrile (4)**

After being left to reflux for four hours, compound 1 (0.256 g, 1 mmol) and chloroacetone (0.11 g, 1.2 mmol) in five milliliters of ethanol and a few drops of triethylamine were combined and chilled. A pale brown powder was obtained by filtering out the precipitated solid product, washing it with ethanol, drying it, and then recrystallizing it from ethanol (yield: 65%). m.p. over 300 °C. IR (KBr, ν, cm^−1^): 3427 (NH, str), 3065–2971 (CH for aromatic and aliphatic), 2228 (CN), 1738, 1664 (2 C=O). ^1^H-NMR (DMSO-d_6_, 500 MHz): *δ* = 2.2 (s, 3H, CO–CH_3_), 3.33 (d, 1H, CH(CN)–CH(Ar), 4.16 (s, 2H, S–CH_2_–CO), 5.297 (s, 1H, methane–CH–CN, 8.087, 8.100 (d, 4H, Aromatic) ppm. Anal. Calcd. for C_15_H_12_N_4_ O_2_S (312.35): C, 57.68; H, 3.87; N, 17.94; S, 10.26.

**4-Amino-8-(4-cyanophenyl)-6-oxo-7,8-dihydro-2H,6H-pyrimido[2,1-b][1,3]thiazine-7-carbonitrile (5)**

Acrylonitrile (0.53 g, 10 mmol) was added to a compound 1 (2.56 g, 10 mmol) solution in [(20 ml) pyridine or nano Mn_3_O_4_ (0.079 g, 1 mmol)], and the reaction mixture was refluxed. The reaction mixture was placed into ice water when it had cooled to room temperature. The solid result was formed by acidifying it with hydrochloric acid (6 ml, 30% soln.) when pyridine was employed. It was then filtered off, cleaned with water, dried, and recrystallized from ethanol to produce a yellow powder (yield: 45–70%) m.p. 240–245 °C. IR (KBr, ν, cm^−1^): 3157–3491 (NH_2_, str), 3085–2870 (CH for aromatic and aliphatic), 2230 (CN), 1678 (C=O). ^1^H-NMR (DMSO-d_6_, 500 MHz): *δ* = 2.49 (*d*, 1*H*, CH(CN)–CH(Ar), 3.1 (s, 1H, methane–CH(CN)), 3.64 (*d*, 2*H,* S–CH_2_–CH) 4.3 (t, 1H, S–CH_2_CH), 7.85, 7.86 (*d*, 4*H*, Aromatic), and 8.9 (s, 2*H*, NH_2_) ppm*. Anal. Calcd.* for *C*_*15*_*H*_*11*_*N*_*5*_*OS (309.35): C, 58.24; H, 3.58; N, 22.64; S, 10.36.*

**7-(4-Cyanophenyl)-3,5-dioxo-2,3,6,7-tetrahydro-5H-thiazolo[3,2-a]pyrimidine-6-carbonitrile (6)**

After allowing compound 1 (0.256 g, 1 mmol) and ethyl chloroacetate (0.1 g, 1.2 mmol) to reflux for 6 h in ethanol (5 ml) with droplets of triethylamine, the reaction mixture was cooled. To get a pale-yellow powder, the precipitated solid product was filtered out, cleaned with ethanol, dried, and recrystallized from ethanol. (yield: 55%) m.p. over 265–270 °C. IR (KBr, ν, cm^−1^): 3131–2966 (CH for aromatic and aliphatic), 2229 (CN), 1675, 1615 (2 C=O). ^1^H-NMR (DMSO-d_6_, 500 MHz): δ = 2.49 (d, 1H, CH(CN)–CH(Ar) 3.36 (s, 1H, methane–CH(CN)), 3.99 (s, 2H, CH_2_ thiazole ring) 7.739, 7.951 (d, 4H, CH Aromatic) ppm. Anal. Calcd. for C_14_H_8_N_4_O_2_S (296.30): C, 56.75; H, 2.72; N, 18.91; S, 10.82.

**(E)-2-benzylidene-7-(4-cyanophenyl)-3,5-dioxo-2,3,6,7-tetrahydro-5H-thiazolo[3,2-a]pyrimidine-6-carbonitrile (7)**

A combination of acetic acid and acetic anhydride (1:1), compound 1 (0.256 g, 1 mmol), chloroacetic acid (0.113 g, 1.2 mmol), benzaldehyde (0.127 g, 1.2 mmol), and anhydrous sodium acetate (0.328 g, 4 mmol), was heated under reflux for seven hours. The resulting solid was collected, repeatedly cleaned with ethanol and acetic acid, dried, and recrystallized from ethanol to produce a greenish-yellow powder (yield: 60%) m.p. 270–275 °C. IR (KBr, ν, cm^−1^): 2228 (CN), 1761, 1694 (2 C=O). ^1^H-NMR (DMSO-d_6_, 500 MHz): δ = 2.49 (d, 1H, CH(CN)–CH(Ar) 3.35 (s, 1H, methine–CH(CN)), 4.5 (s, 1H, C=CH benzylidene), 7.771, 7.787 (m, 9H, Aromatic) ppm. Anal. Calcd. for C_21_H_12_N_4_O_2_S (384.41):C, 65.61; H, 3.15; N, 14.57; S, 8.34.

**2-(Benzo[d]thiazol-2-ylamino)-4-(4-cyanophenyl)-6-oxo-1,4,5,6-tetrahydro pyrimidine-5-carbonitrile (8)**

Compound 2 (0.27 g, 1 mmol) and 2-aminobenzothiazole (0.15 g, 1 mmol) were combined with 10 ml of ethanol, a few drops of triethylamine, and refluxed for five hours before cooling to room temperature. The reaction mixture was then added into frozen water. To get a pale brown powder, the precipitated solid product was filtered out, cleaned with ethanol, dried, and recrystallized from ethanol (yield: 70%) m.p. 285–290 °C. IR (KBr, ν, cm^−1^): 3448, 3422 (2 NH, str), 3099–2926 (CH for aromatic and aliphatic), 2230 (CN), 1676 (C=O). ^1^H-NMR (DMSO-d_6_, 500 MHz): δ = 3.06 (d, 1H, CH(CN)–CH (Ar)), 3.48 (s, 1H, methane–CH(CN)), 7.85–7.47 (m, 8H, CH Aromatic), 9.92, 11.95 (s, 2H, 2 NH, str) ppm. Anal. Calcd. for C_19_H_12_N_6_OS (372.08): C, 61.28; H, 3.25; N, 22.57; S, 8.61.

**4-(4-Cyanophenyl)-6-oxo-2-(2-phenylhydrazine)-1,4,5,6-tetrahydro pyrimidine-5-carbonitrile (9)**

Compound 2 (0.27 g, 1 mmol) and phenylhydrazine (0.108 g, 1 mmol) were combined with 10 ml of ethanol, a few drops of triethylamine, and refluxed for four hours before cooling to room temperature. The reaction mixture was then added to freezing water. To get a pale brown powder, the precipitated solid product was filtered out, cleaned with ethanol, dried, and recrystallized from ethanol. (yield: 55%) m.p. over 300 °C. IR (KBr, ν, cm^−1^): 3449 (NH, str), 2224 (CN), 1669 (C=O). ^1^H-NMR (DMSO-d_6_, 500 MHz): δ = 2.62 (d, 1H, CH(CN)–CH(Ar)), 3.37 (s, 1H, methane–CH(CN)), 7.693–7.624 (m, 9H, CH Aromatic), 8.205–8.189 (d, 2H, 2NH, str), 8.002 (s, 1H, NH, str) ppm. Anal. Calcd. for C_18_H_14_N_6_O (330.35): C, 65.44; H, 4.27; N, 25.44.

### Computer prediction of biological activity of synthetic compounds

Based on its structural formula, a computer tool such as the PASS and Swiss ADME web resources may be used to assess the expected profile of biological activity of a drug-like organic molecule whose molecular mass spans from 50 to 1250 Da. The assessment is established on a review of the structure–activity interactions for a sizable training set comprising chemical probes, drug substances, drug candidates at different phases of preclinical and clinical development, pharmaceutical agents, and compounds for which specific toxicity data is available.

### Drug-likeness and oral bioavailability analysis of synthetic compounds using Swiss ADME web resources

Early in the drug development progression, it is crucial to analyze the pharmacokinetic characteristics of candidate medications. To represent the excretion of drugs, the total clearance model and renal OCT2 substrate were utilized^27,29^.

### Docking study

The software of the molecular operating environment was used to accomplish the molecular modeling for the highest active compounds, which was outlined using ChemDraw Ultra 12.0, as confirmed by Cousins (2011) and Cheema et al. (2024)^27,30,31^.

For the modeling of the target protein, the identity of appropriate homologous template structures was accomplished by means of automated homology modeling pipeline SWISS-MODEL (achieved by Swiss Institute of Bioinformatics). In place of improvement of the outcomes, London DG force and force field energy were used. All feasible minimizations were made in anticipation of a root mean square deviation (RMSD) gradient of 0.1 kcal mol^−1^ Å^−1^ via MMFF 94× (Merck molecular force field 94×) and the partial charges were spontaneously deliberated. The ligand binding affinity was evaluated by means of the scoring function; dock function (S, Kcal/mol) created by the MOE 2022 software. From the protein data bank, the X-ray crystal structure of the enzyme’s (PDB ID: 2FV5, resolution: 2.10 Å) was downloaded in the PDB format. For docking investigations, the enzyme was prescribed after the following arrangement: (i) The water was removed from the protein (ii) Hydrogen atoms (H) with their standard geometry, were added to the structure then reconnect the bonds broken and fixing the potential. (iii) For the large site search in the enzyme structure, MOE Alpha Site Finder was used, and dummy atoms were generated from the resulting alpha spheres. (iv) Analyzing the ligand's interaction with the active sites of the amino acids. The active ligands with the highest Docking Score have the most negative values. All docking processes and scoring were recorded based on established criteria. For docking, the Triangle Matcher placement method and the London dG score tool were employed^21,32–41^.

## Conclusions

Alzheimer's disease (AD), which affects a vast population globally, is defined by the elderly's loss of memory and learning capacity, our findings contribute to the body of knowledge on drugs that may someday have pharmacokinetic properties, making them prospective therapeutic options for Alzheimer's disease treatment.

The Biginelli synthesis of 4-cyanobenzaldehyde, ethyl cyanoacetate, and thiourea in the presence of Mn_3_O_4_ nanoparticle provides 4-(4-cyanophenyl)-6-oxo-2thioxohexahydropyrimidine-5-carbonitrile (1), which is a cost-effective and environmentally beneficial procedure. This multi-part method is non-toxic and safe. Compounds were created by the reaction of compound (1) with various reagents. Together, these ground-breaking studies were used to look into synthetic compounds in preliminary ways. Conventional docking studies were utilized to evaluate the best docked ligands, allowing us to discover the compounds' binding mechanisms. The binding energies of the drug-target interactions are important in determining how well the drug binds to the target macromolecule.

### Supplementary Information

Supplementary Information.

## Data Availability

All data generated or analysed during this study are included in this published article [and its supplementary information files].
