# Supplementary material for: Pharmacokinetic and molecular docking studies to pyrimidine drug using Mn3O4 nanoparticles to explore potential anti-Alzheimer activity
Source: Sci Rep. 2024 Jul 4;14:15436. doi: 10.1038/s41598-024-65166-2 (PMC11224222; doi:10.1038/s41598-024-65166-2)
Supplement: Supplementary file 1 — Supplementary Information. [file 41598_2024_65166_MOESM1_ESM.docx]

**Spectral Analysis**

4-(4-cyanophenyl)-6-oxo-2-thioxohexahydropyrimidine-5-carbonitrile (1):

**IR Spectrum of (1):**


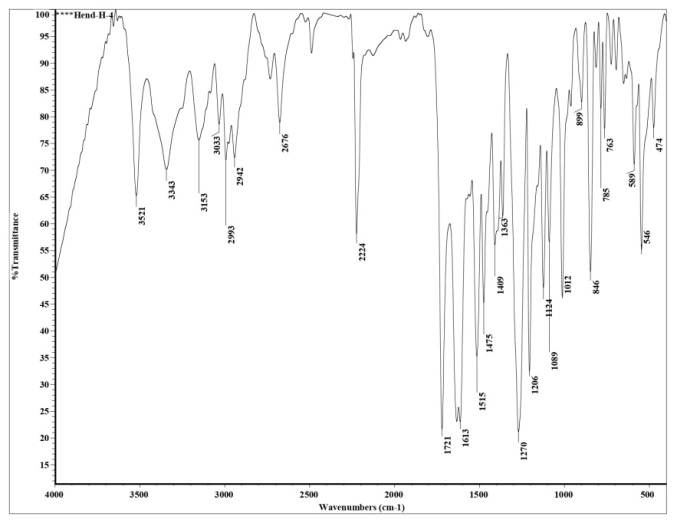

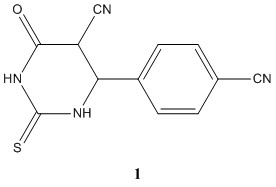


**^1^H-NMR Spectrum of (1):**


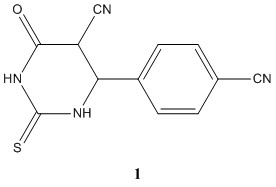


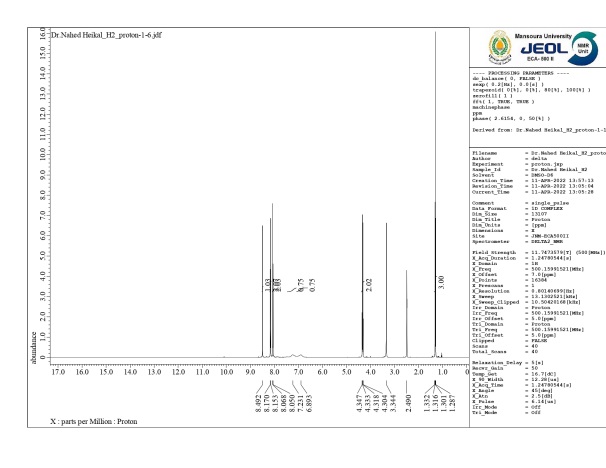

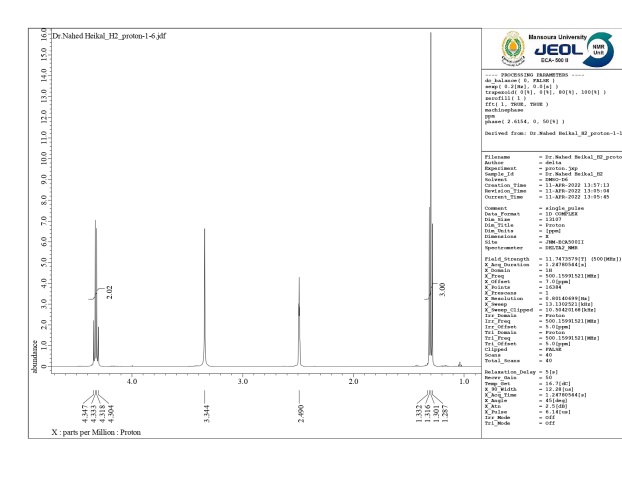

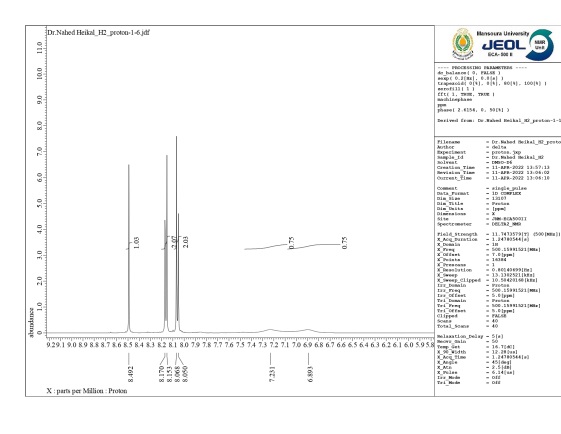


**^13^C-Spectrum of (1):**


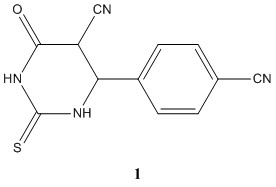


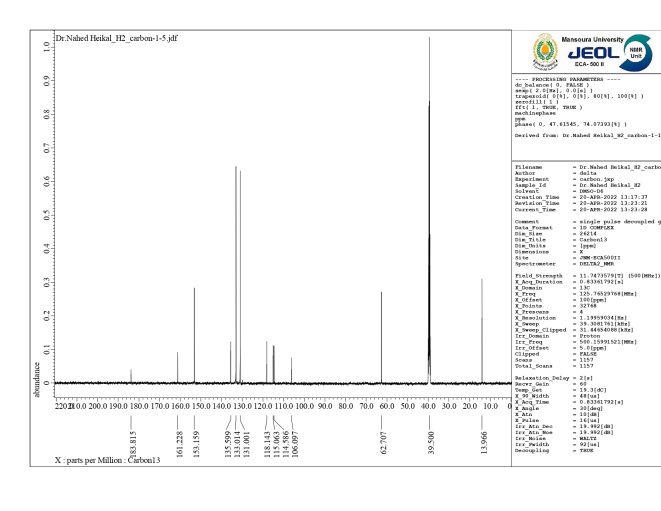

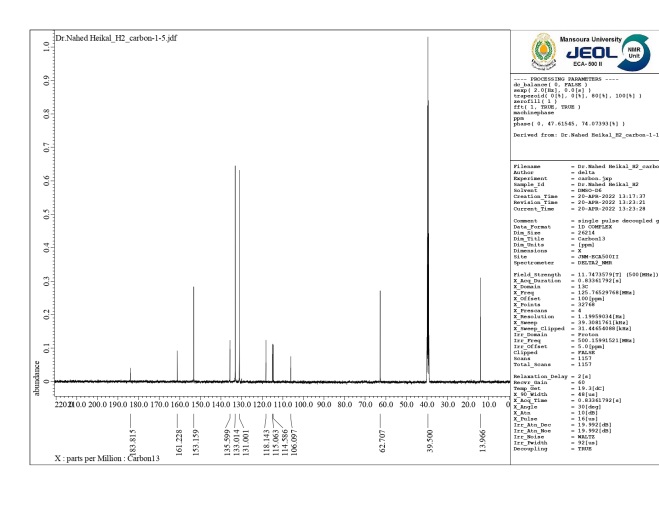


**4-(4-Cyanophenyl)-2-(methylthio)-6-oxo-1,4,5,6-tetrahydropyrimidine-5-carbonitrile (2):**

IR Spectrum of (2):

**
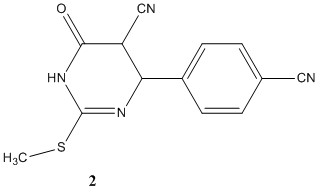
**
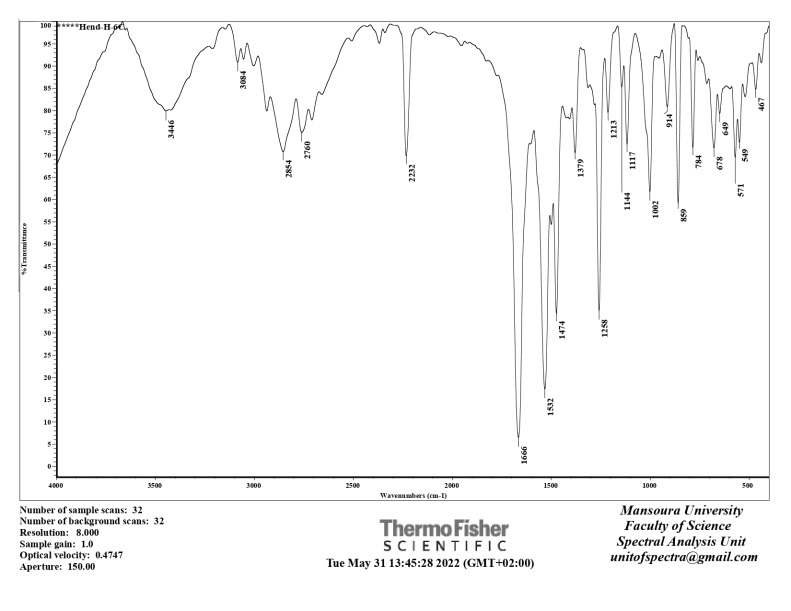


^1^H-NMR Spectrum of (2):

**
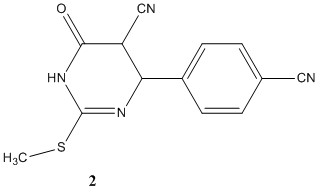
**


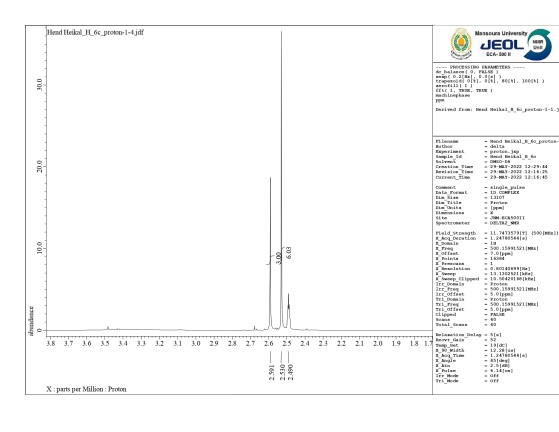

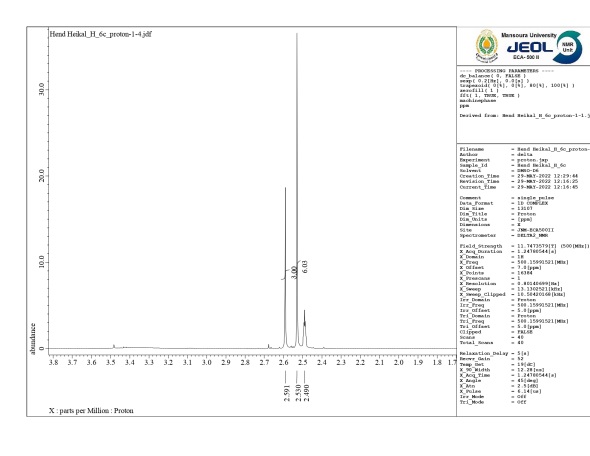

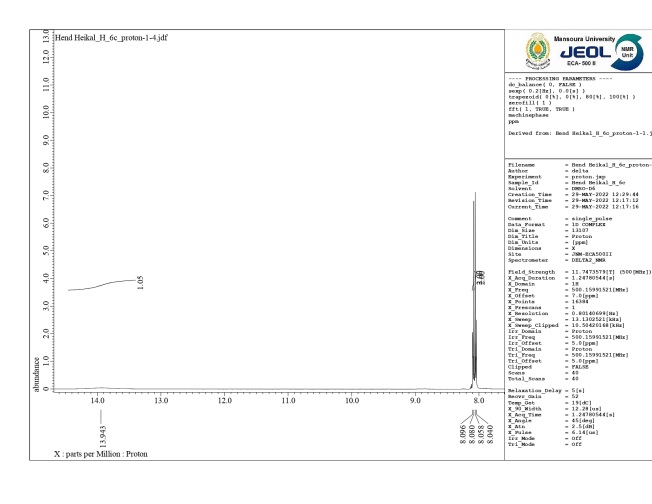


^13^C-Spectrum of (2):

**
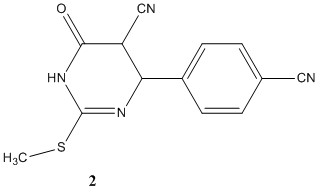
**

^
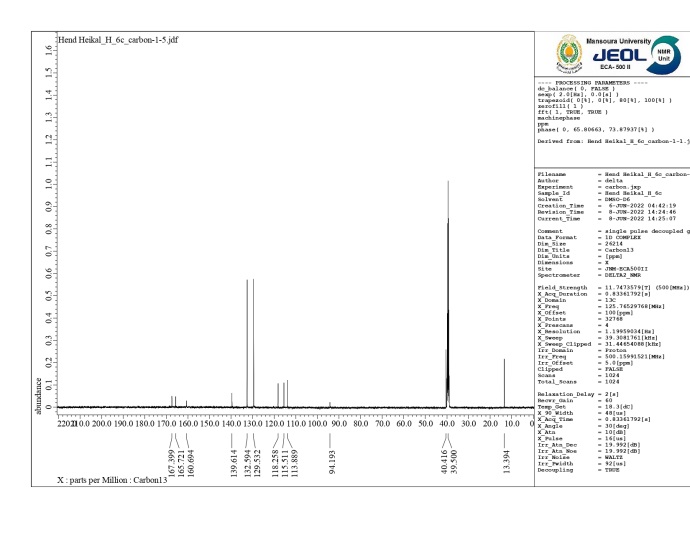
^

2-((cyanomethyl)thio)-4-(4-cyanophenyl)-6-oxo-1,4,5,6-tetrahydropyrimidine-5-carbonitrile **( 3 )**:

**IR Spectrum of (3):**


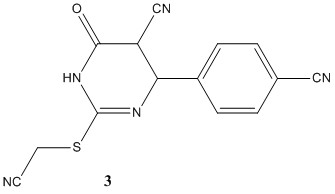


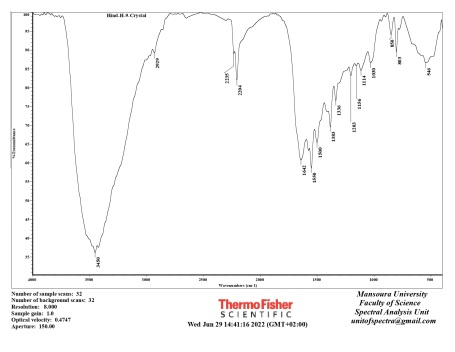


**^1^H-NMR Spectrum of (3):**


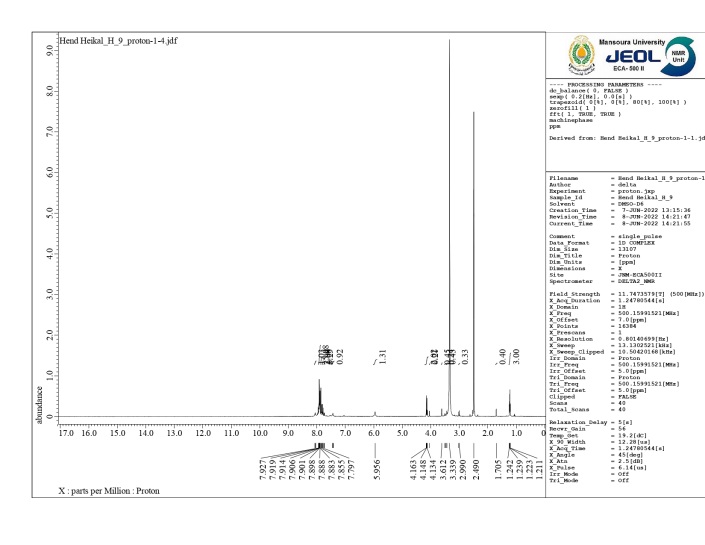

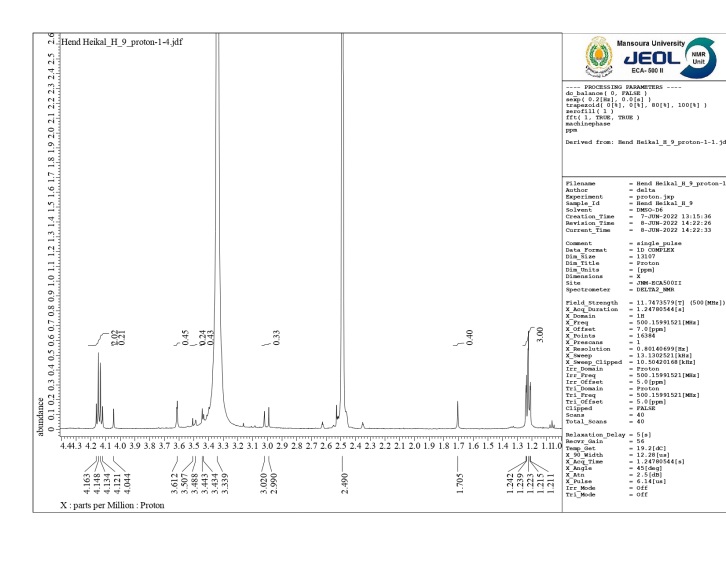

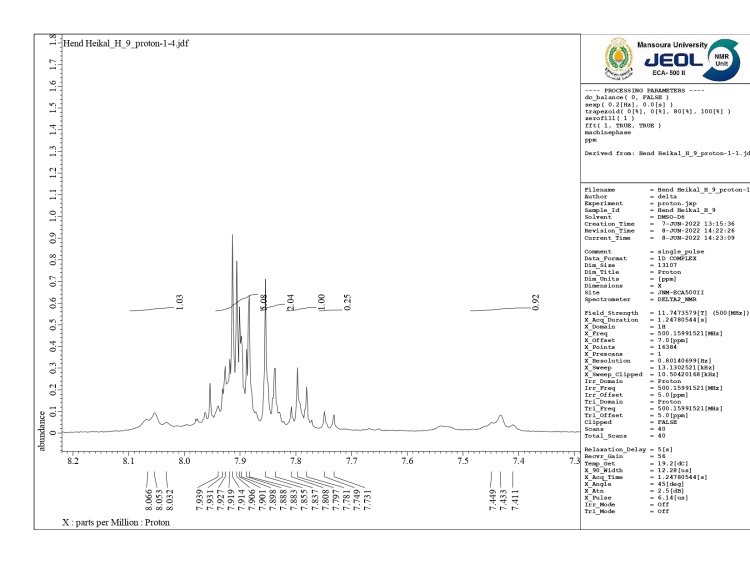


4-(4-cyanophenyl)-6-oxo-2-((2-oxopropyl)thio)-1,4,5,6-tetrahydropyrimidine-5-carbonitrile **(4)**:

**IR Spectrum of (4):**

**
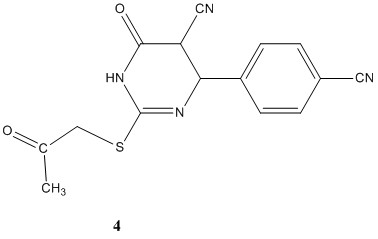
**

**
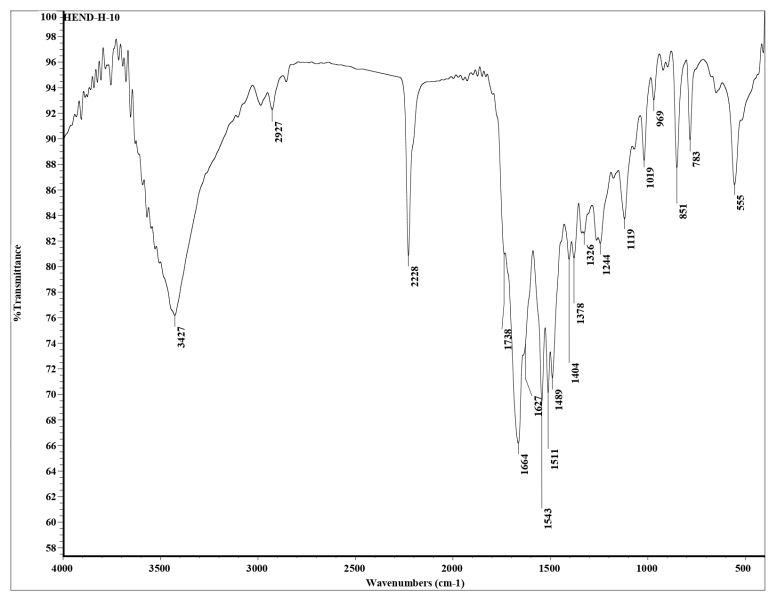
**

**^1^H-NMR Spectrum of (4):**

**
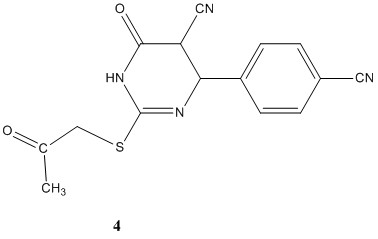
**
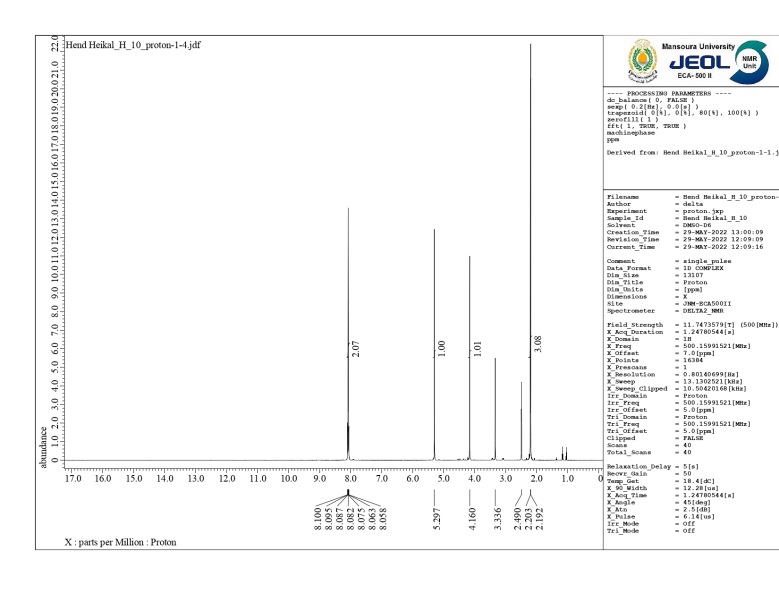

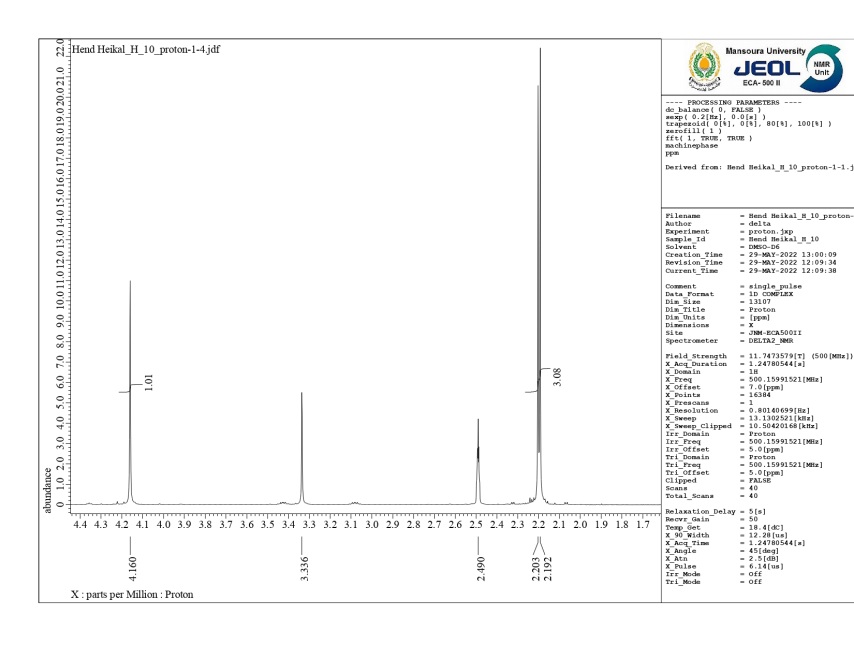

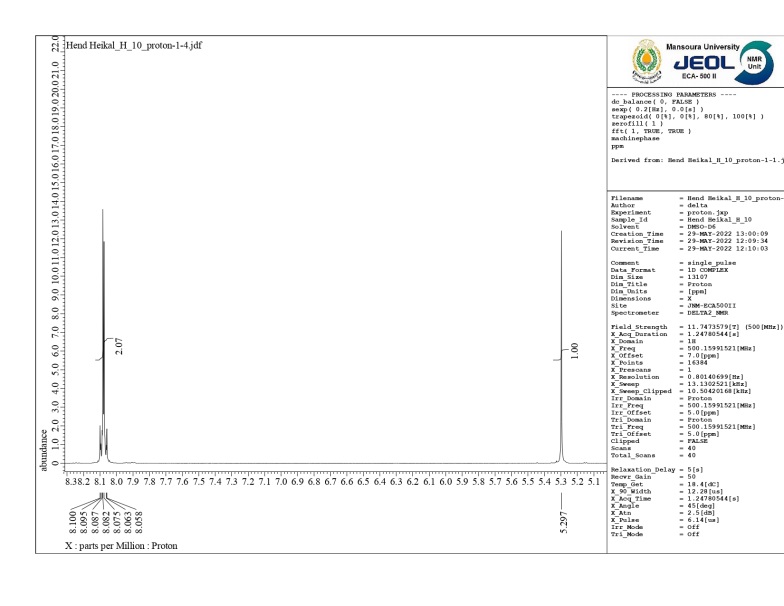


**4-Amino-8-(4-cyanophenyl)-6-oxo-7,8-dihydro-2H,6H-pyrimido[2,1-b][1,3]thiazine-7-carbonitrile (5):**

**IR Spectrum of (5):**

**
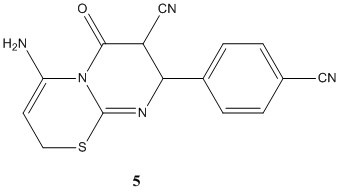
**

**
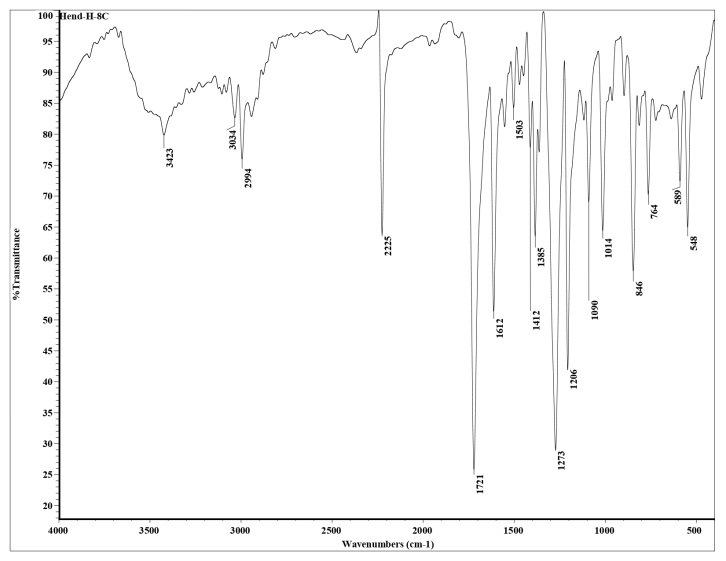
**

**^1^H-NMR Spectrum of (5):**

**
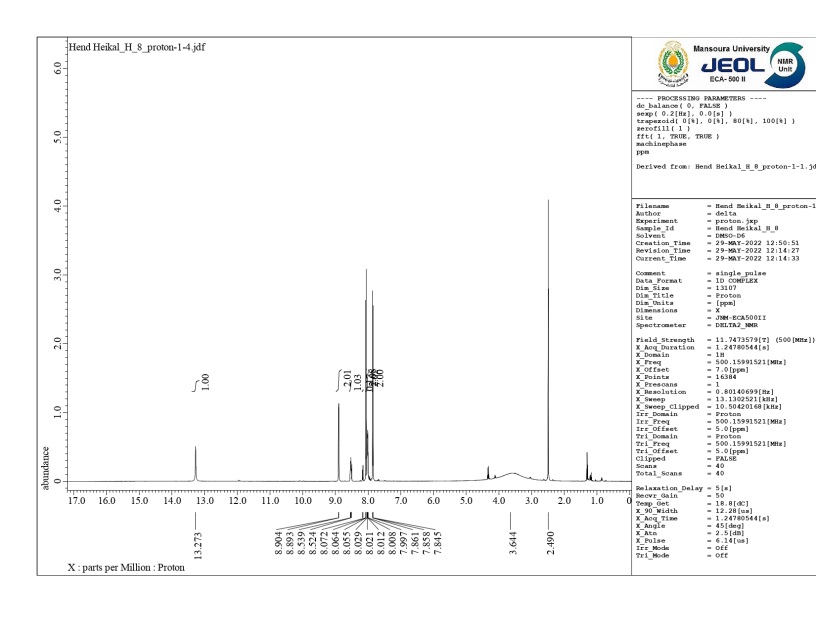

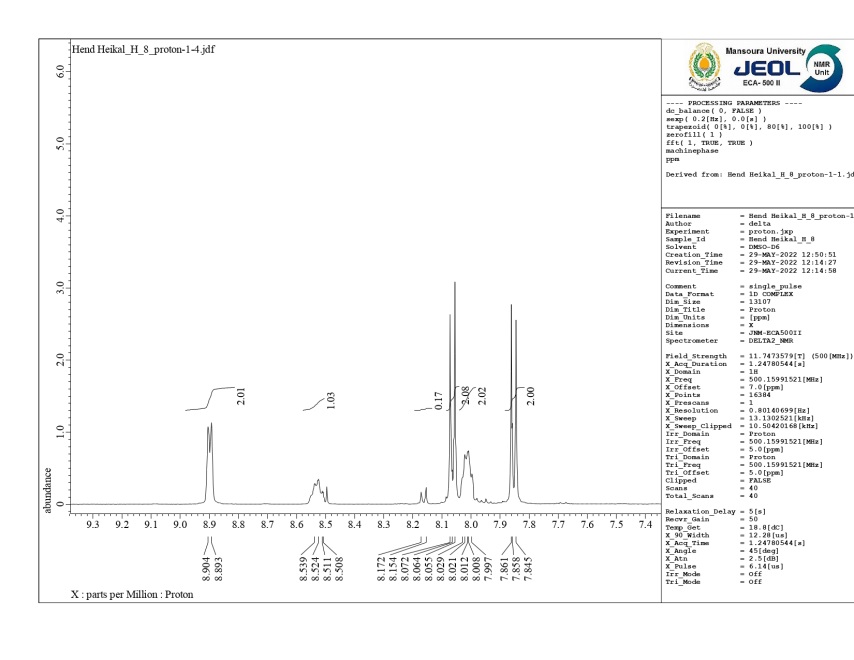
**

**7-(4-Cyanophenyl)-3,5-dioxo-2,3,6,7-tetrahydro-5H-thiazolo[3,2-a]pyrimidine-6-carbonitrile (6):**

**IR Spectrum of (6):**

**
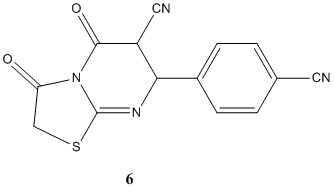
**

**
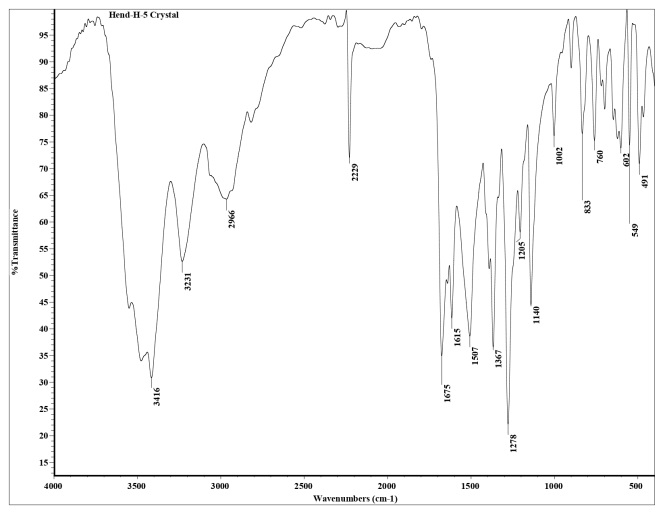
**

**^1^H-NMR Spectrum of (6):**

**
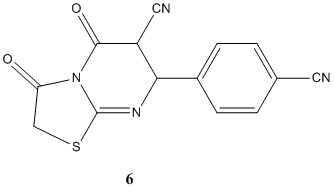

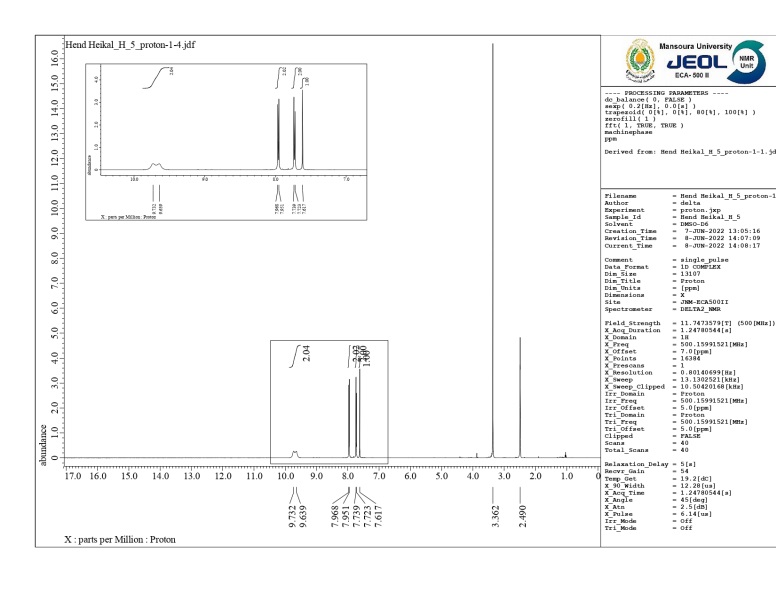
**

**(E)-2-benzylidene-7-(4-cyanophenyl)-3,5-dioxo-2,3,6,7-tetrahydro-5H-thiazolo[3,2-a]pyrimidine-6-carbonitrile (7):**

**IR Spectrum of (7):**


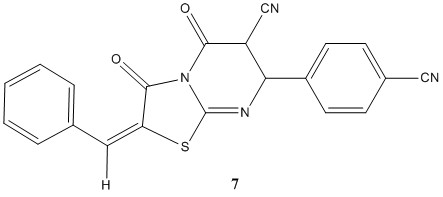


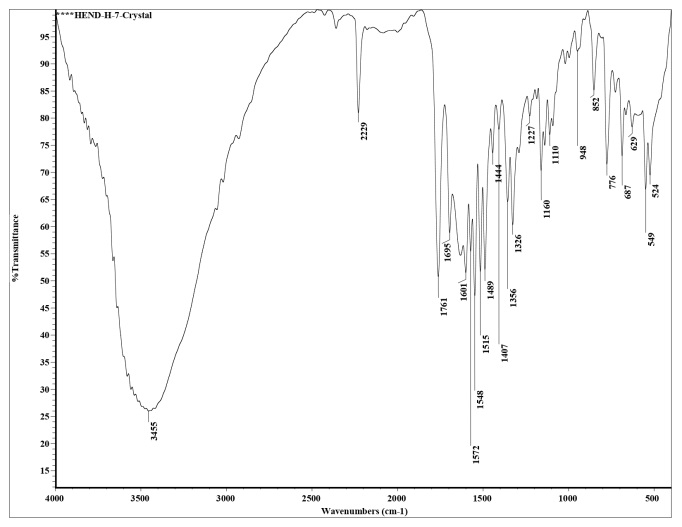


**^1^H-NMR Spectrum of (7):**


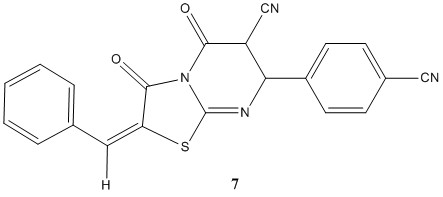
**
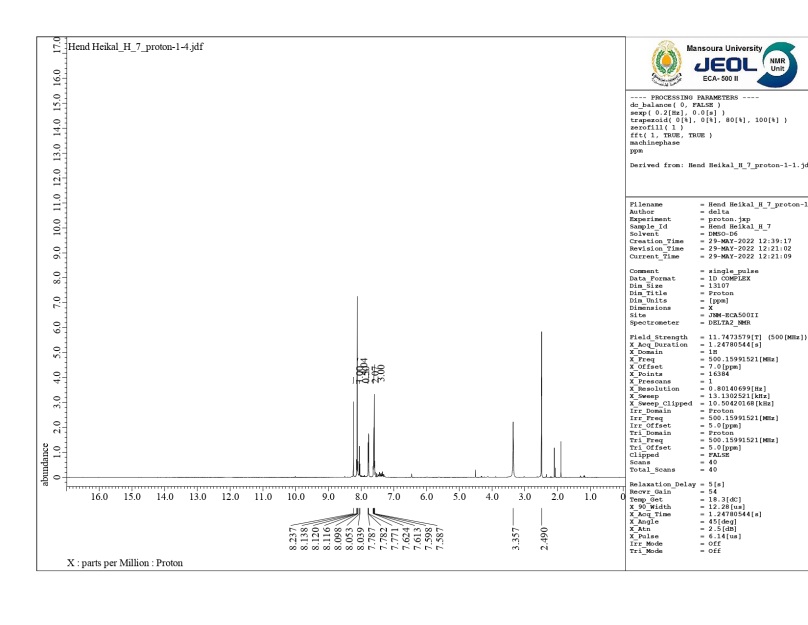
**

**2-(Benzo[d]thiazol-2-ylamino)-4-(4-cyanophenyl)-6-oxo-1,4,5,6-tetrahydropyrimidine-5-carbonitrile ( 8 ):**

**IR Spectrum of (8):**

**
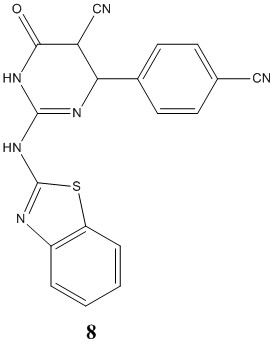
**

**
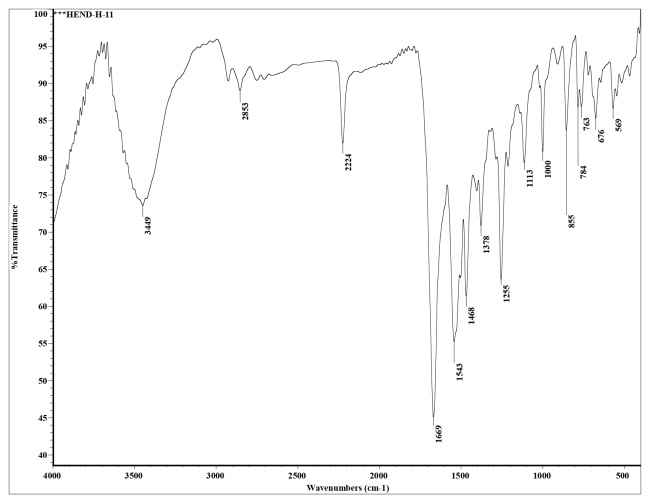
**

**^1^H-NMR Spectrum of (8):**

**
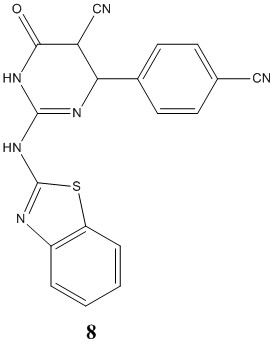

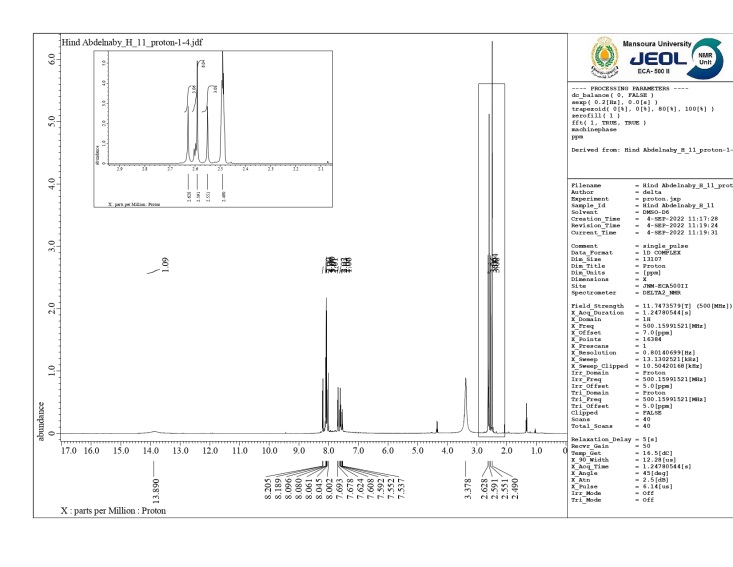

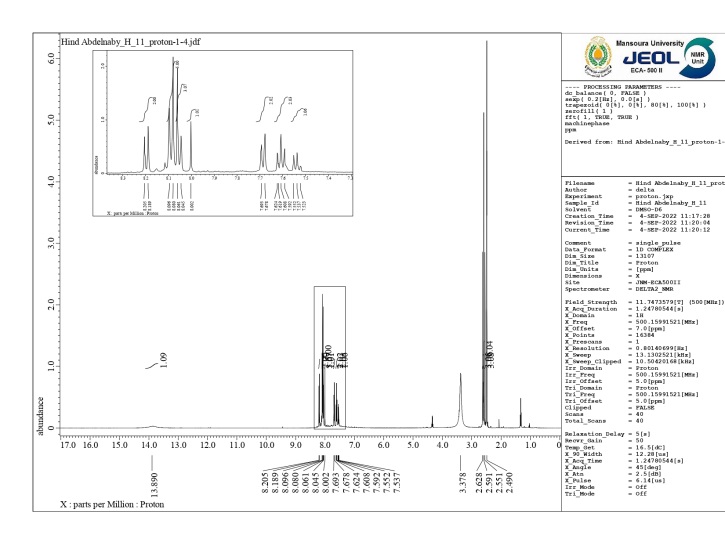
**

**4-(4-Cyanophenyl)-6-oxo-2-(2-phenylhydrazineyl)-1,4,5,6-tetrahydropyrimidine-5-carbonitrile ( 9 ):**

**IR Spectrum of (9):**

**
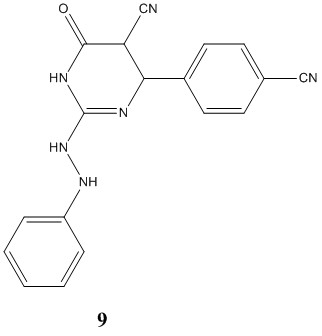
**

**
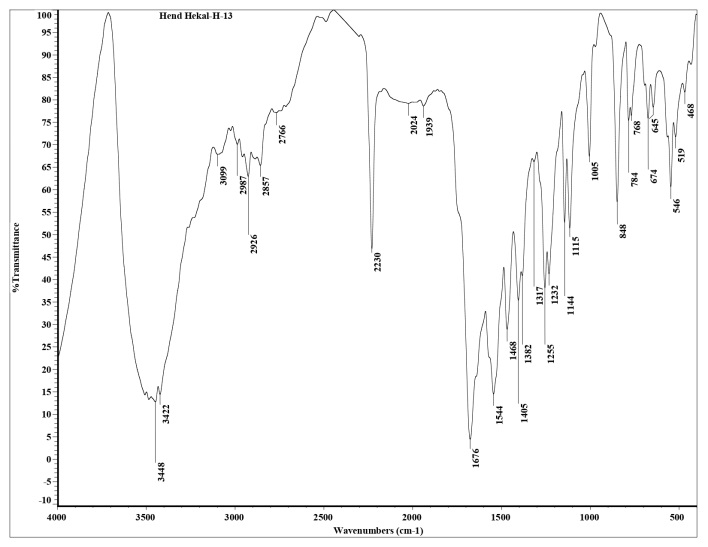
**

**^1^H-NMR Spectrum of (9):**

**
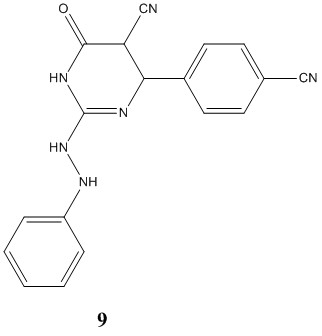

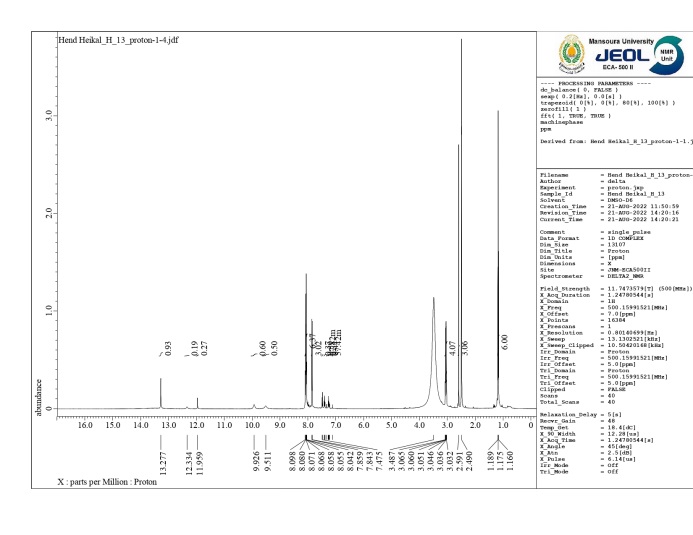

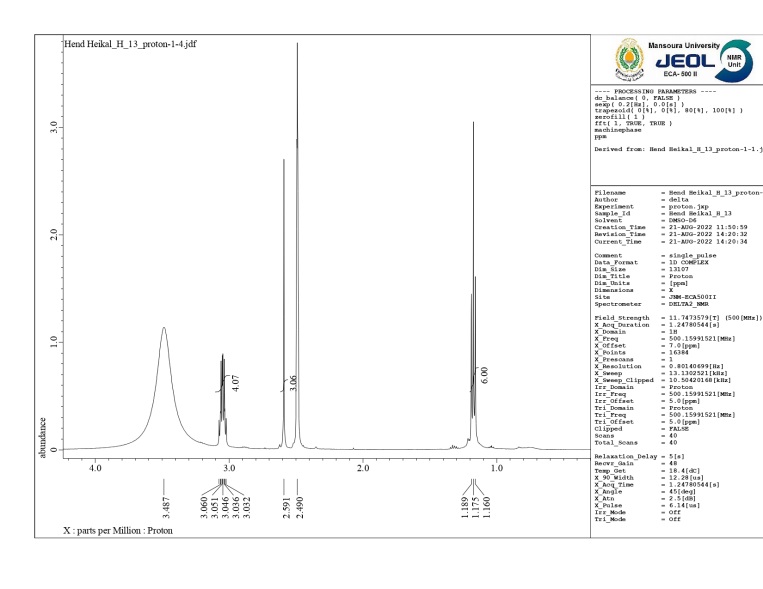

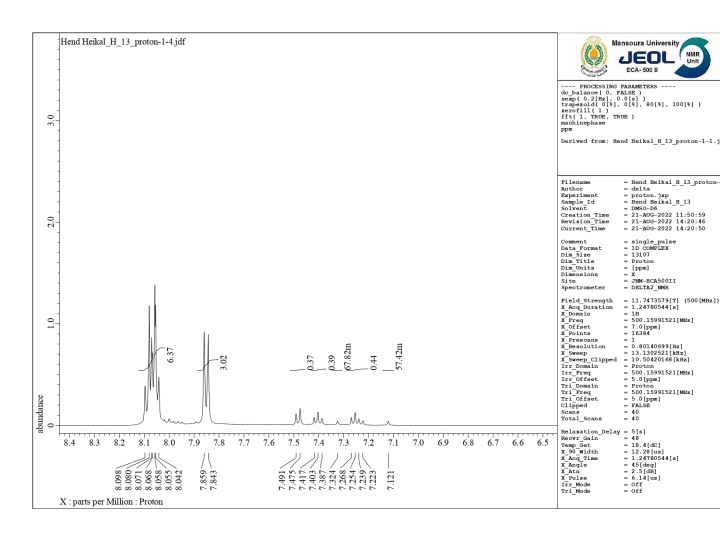

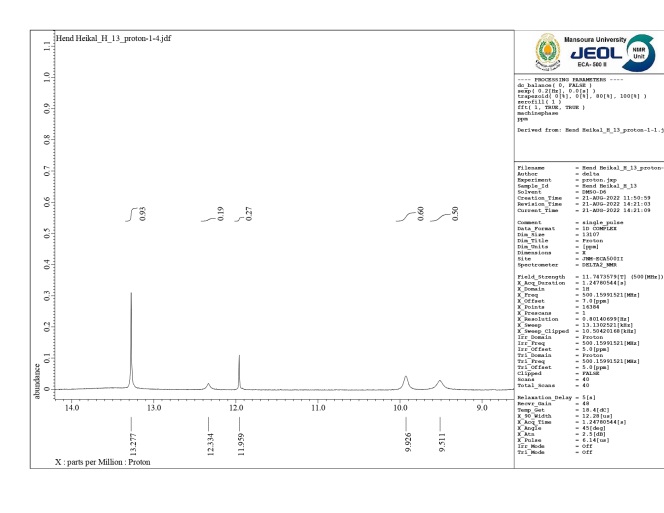
**
